# Pyrroline-5-carboxylate reductase 1 reprograms proline metabolism to drive breast cancer stemness under psychological stress

**DOI:** 10.1038/s41419-023-06200-5

**Published:** 2023-10-16

**Authors:** Bai Cui, Bin He, Yanping Huang, Cenxin Wang, Huandong Luo, Jinxin Lu, Keyu Su, Xiaoyu Zhang, Yuanyuan Luo, Zhuoran Zhao, Yuqing Yang, Yunkun Zhang, Fan An, Hong Wang, Eric W.-F. Lam, Keith W. Kelley, Ling Wang, Quentin Liu, Fei Peng

**Affiliations:** 1 Institute of Cancer Stem Cell, Dalian Medical University, Dalian, China; 2 State Key Laboratory of Oncology in South China, Cancer Center, Sun Yat-sen University, Guangzhou, China; 3 Department of Oncology, the First Affiliated Hospital of Dalian Medical University, Dalian, China; 4 CAS Key Laboratory of Separation Science for Analytical Chemistry, Dalian Institute of Chemical Physics, Chinese Academy of Sciences, Dalian, China; 5 Department of Pathology, The Second Hospital of Dalian Medical University, Dalian, China; 6 Department of Orthopaedics, The Central Hospital of Dalian University of Technology, Dalian, China; 7 Department of Surgery and Cancer, Imperial College London, London, UK; 8 Department of Pathology, College of Medicine and Department of Animal Sciences, College of ACES, University of Illinois at Urbana-Champaign, Urbana, Illinois USA

**Keywords:** Breast cancer, Cancer stem cells

## Abstract

Cancer stem-like cells (CSCs) contribute to cancer metastasis, drug resistance and tumor relapse, yet how amino acid metabolism promotes CSC maintenance remains exclusive. Here, we identify that proline synthetase PYCR1 is critical for breast cancer stemness and tumor growth. Mechanistically, PYCR1-synthesized proline activates cGMP-PKG signaling to enhance cancer stem-like traits. Importantly, cGMP-PKG signaling mediates psychological stress-induced cancer stem-like phenotypes and tumorigenesis. Ablation of PYCR1 markedly reverses psychological stress-induced proline synthesis, cGMP-PKG signaling activation and cancer progression. Clinically, PYCR1 and cGMP-PKG signaling components are highly expressed in breast tumor specimens, conferring poor survival in breast cancer patients. Targeting proline metabolism or cGMP-PKG signaling pathway provides a potential therapeutic strategy for breast patients undergoing psychological stress. Collectively, our findings unveil that PYCR1-enhanced proline synthesis displays a critical role in maintaining breast cancer stemness.

## Introduction

Triple-negative breast cancers (TNBCs) account for ~15% of patient diagnoses [1]. As the lack of well-defined molecular drivers and effective targeted therapies, TNBC is associated with advanced stage, high recurrence rates and poor survival [2, 3]. Accumulating studies have documented that chemoresistance and the poor prognosis of TNBC patients are due to the presence of a small subpopulation of cancer cells with stem cell properties, referred to as cancer stem-like cells (CSCs) [4–6]. CSCs exhibit enhanced self-renewal capacity, pluripotency and multilineage differentiation ability to generate various cell populations that ultimately form tumors [7, 8]. Importantly, metabolic reprograming represents a fundamental hallmark of CSCs and is responsible for orchestrating both tumor progression and recurrence [9]. Emerging evidence has shown that dysfunctional glucose and lipid metabolism play crucial roles in cancer stem cell maintenance through energy dependent metabolic rewiring [10]. For example, our study has reported that lactate dehydrogenase A (LDHA)-catalyzed lactate induces tumor acidic microenvironment to strengthen the binding capacity between USP28 and MYC, leading to MYC-driven breast cancer stem-like traits [11]. JAK/STAT3 transactivates CPT1B to trigger fatty acid oxidation, contributing to breast CSC self-renewal and chemoresistance [12]. Hence, these findings reveal that understanding the metabolic reprogramming of CSCs is critical for devising improved therapies for diverse cancers.

Amino acids as basic protein units and metabolic regulators are involved in tumor development [13, 14]. Recent studies show that amino acids rewiring is associated with leukemic stem-like cell (LSC) functions. For example, LSCs exhibit higher levels of amino acids, and that amino acid metabolism for OXPHOS is essential for survival of the de novo LSC in AML [15]. PRMT7-deficiency reduces glycine decarboxylase to promote glycine metabolism reprograming, generating methylglyoxal to dampen LSCs [16]. In addition, CD9 functioning as a marker of PDAC tumor-initiating cells promotes plasma membrane localization of glutamine transporter ASCT2 to enhance glutamine uptake, contributing to organoid formation capability and tumor grafts in vivo [17]. Glutamine catabolism also contributes to the maintenance of prostate CSCs through the regulation of alpha-ketoglutarate (α-KG)-dependent chromatin-modifying dioxygenase [18]. Although amino acids are validated as early diagnostic biomarkers for breast cancer [19], the unique amino acid metabolism in regulating breast CSCs remains unclear.

Proline, substantially increased in cancer cells, is essential for the protein synthesis and bioenergetics, leading to the rapid and unlimited cellular proliferation [20]. Furthermore, proline metabolic process is aberrantly upregulated in CSCs [21]. However, it is still unclear whether proline metabolism determines the fate of CSCs. Catalyzing NAD(P)H-dependent conversion of delta1-pyrroline-5-carboxylate (P5C) to proline, Pyrroline-5-carboxylate reductase 1 (PYCR1) is a key enzyme in the final step of proline biosynthesis [22]. PYCR1, overexpressed in breast tumors, is significantly associated with tumor growth, advanced grades and poor survival of patients [23]. Functional genomics has confirmed that PYCR1 is required for malignant proliferation of breast tumors [24]. Specially, knockdown of PYCR1 inhibits breast cancer cell invasion by blocking MMP9 activity [25]. Ablation of PYCR1 attenuates oncogenic effect of MCF10A H-Ras^V12^ breast transformed cells by impairing P5C recycling [26]. As high metastatic capacity and oncogenicity are major characteristics of CSCs [27], whether and how PYCR1-enhanced proline controls cancer stem-like properties remain to be determined.

Cancer patients often suffer from chronic psychological stress after undergoing a series of stressful events, including the diagnosis, treatment and multiple cancer-related complications [28]. Emerging epidemiological studies have demonstrated a remarkable positive association between psychological stress and cancer incidence, progression and the poor outcome of cancer treatments [29]. In the case of breast cancer, a systematic meta-analysis of 282,203 patients reveals that depression and anxiety in breast cancer patients predict a higher risk for both recurrence and mortality [30]. Our previous study unveils that psychological stress promotes glycolysis rewiring to enhance breast stem-like properties [11]. This finding indicates that the metabolic reprogramming is critical for psychological stress-derived breast malignancy. In addition, abnormal proline metabolism exacerbates depressive disorder [31] and exhibits association with psychoneurological symptoms of breast cancer patients [32]. However, whether proline metabolism mediates stress-induced tumor development and cancer stem-like phenotypes has yet to be determined.

Here we identify that PYCR1-induced proline biosynthesis enhances TNBC stem cell-like properties and tumor growth. Moreover, PYCR1-synthesized proline activates cGMP-PKG signaling pathway in CSC maintenance. Furthermore, cGMP-PKG signaling facilitates psychological stress-induced cancer stemness and progression. Ablation of PYCR1 reverses psychological stress-induced cGMP-PKG signaling activation, stem-like traits and cancer progression. In the clinic, the level of PYCR1-cGMP-PKG axis is enriched in tumor specimens from TNBC patients and confers poor prognosis. Taken together, these findings uncover a PYCR1-cGMP-PKG signaling axis that is critically involved in regulation of psychological stress-mediated CSC maintenance and tumor growth. Together these findings suggest new and attractive targets in the identification and treatment of TNBCs.

## Results

### PYCR1 is critical for breast cancer stem-like cell maintenance

**Fig. 1:**
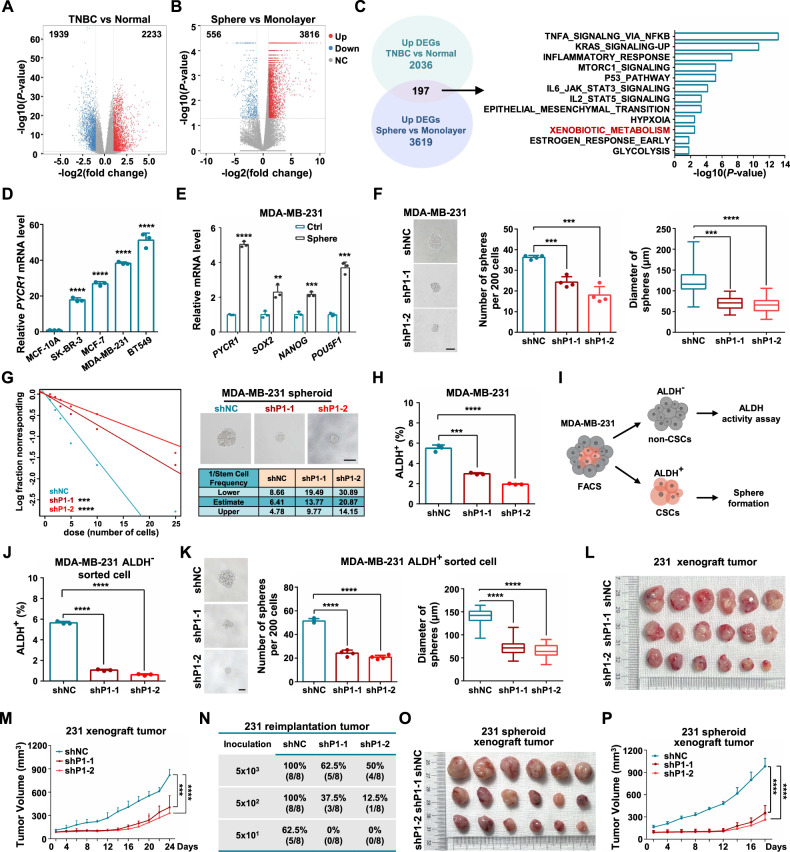
PYCR1 is required for breast cancer stem-like cell maintenance. **A**, **B** Volcano plots represent DEGs comparing TNBC tumor tissues with adjacent normal tissues (SRP157974) (**A**) and DEGs comparing spheroids with monolayer MDA-MB-231 cells (GSE116180) (**B**). The number of significantly variant genes (FC > 2, *P* < 0.05) was shown. Vertical dashed lines indicate cut-off of FC (2), whereas the horizontal dashed lines indicate cut-off of *P*-value (0.05). **C** The GSEA hallmark gene sets analysis of overlapped DEGs upregulated in TNBC samples and sphere MDA-MB-231 cells. **D** Relative mRNA levels of *PYCR1* in mammary epithelium and breast cancer cell lines (*n* = 3). **E** Relative mRNA levels of *PYCR1*, *SOX2*, *NANOG* and *POU5F1* in spheroids and MDA-MB-231 cells (*n* = 3). **F** Sphere formation ability was analyzed following ablation of PYCR1 (shP1-1, shP1-2) in MDA-MB-231 cells. The representative images were presented (scale bar = 100 μm) and the number and diameter of spheroids were measured and counted (*n* = 4). **G** Left: second generation Extreme Limiting Dilution Analysis was performed by plating MDA-MB-231 spheroids (from F). Top right: The representative sphere images are shown. Scale bars, 100 μm. Bottom right: Stemness frequency illustration of the cells with the upper and lower 95% confidence intervals meaning that the frequency of one stem cell in cancer cells. Spheres were counted from 16 replicate wells. **H** Differences of ALDH-positive cells in MDA-MB-231 cells following ablation of PYCR1 (shP1-1, shP1-2) were determined (*n* = 3). **I** Schematic of experimental procedure used to sort ALDH^-^ and ALDH^+^ MDA-MB-231 cells for further assays. **J** Flow cytometry analysis for ALDH-positive cells in sorted ALDH^-^ MDA-MB-231 (shP1-1 and shP1-2) cells after 6 days monolayer culture (*n* = 3). **K** Sphere formation assay was performed in sorted ALDH^-^ MDA-MB-231 shNC, shP1-1 and shP1-2 cells. The representative images were presented (Left, scale bar = 100 μm) and the number (Middle) and diameter (Right) of spheroids were measured and counted (*n* = 4). **L**, **M** Immunodeficient mice (*n* = 6) were subcutaneously inoculated with monolayer shP1-1 and shP1-2 MDA-MB-231 cells (**L**) and tumor volumes were monitored (**M**). **N** Serially diluted tumor cells from xenograft tumors (**L**) were subcutaneously inoculated at 3 different sites into each group of mice. Statistical analysis of tumorigenesis with indicated cell numbers and different treatments is shown (*n* = 8). **O**, **P** Cells from MDA-MB-231 spheroids (Fig. 1F) were subcutaneously inoculated in immunodeficient mice (**O**) (*n* = 6). Tumor volumes were monitored (**P**). FC, fold change. DEGs, differentially expressed genes. GSEA, gene set enrichment analysis. Graph data were presented as mean ± SD. **P < 0.01, ***P < 0.001, ****P < 0.0001. *P*-values were calculated with two-tailed, unpaired Student’s *t*-test.

To identify the regulators involved in triple negative breast cancer (TNBC) stem-like properties, we investigated differentially expressed genes (DEGs) in both TNBC specimens and cancer stem cell (CSC)-enriched spheroids (Supplementary Fig. 1A, B). The results showed that 2,233 genes were upregulated in TNBC specimens compared with normal tissues (Fig. 1A) and 3816 genes were upregulated in spheroids compared with monolayer MDA-MB-231 cells (Fig. 1B). Next, we overlapped the upregulated genes and conducted a Gene Set Enrichment Analysis (GSEA) to identify the altered biological processes. In that, we found that the majority of enriched signaling pathways were regulated to cancer stemness, except for the xenobiotic metabolism (Fig. 1C). Hence, we examined the xenobiotic metabolism-associated genes (*PYCR1*, *KYNU*, *CFB*, *HMOX1* and *HES6*) expressed in both breast cancer cells and in normal mammary epithelial cells. Compared with other genes, the mRNA level of *PYCR1* was evidently higher in breast cancer cells, especially the TNBC cells (Fig. 1D, Supplementary Fig. 1C–F). Moreover, *PYCR1* mRNA levels in spheroids (characterized through their overexpression of stemness-related factors *SOX2*, *NANOG* and *POU5F1*) were substantially higher than those in monolayer MDA-MB-231 cells (Fig. 1E). We further examined the expression of the other PYCR isoforms- *PYCR2* and *PYCR3* [33]. The results showed that mRNA levels of *PYCR2* and *PYCR3* were not evidently increased in TNBC cells (Supplementary Fig. 1G, H). *PYCR2* mRNA level increased but *PYCR3* mRNA level slightly decreased in spheroids (Supplementary Fig. 1I).

Furthermore, PYCR1 stable knockdown cells established by two shRNAs displayed a decrease in protein and mRNA expression levels of the stemness-related factors SOX2, NANOG and β-Catenin (Supplementary Fig. 1J, K). Sphere-formation assays showed a significant reduction in spheroid numbers and diameters in PYCR1-knockdown MDA-MB-231 cells (Fig. 1F). The second generation using the Extreme Limiting Dilution Analysis showed that knockdown PYCR1 significantly decreased sphere formation capacity of spheroid cells (Fig. 1G). The cancer stem cell-associated ALDH^+^ population significantly declined following ablation of PYCR1 in MDA-MB-231 cells (Fig. 1H, Supplementary Fig. 1L). Whereas forced expression of PYCR1 had no effect on the proportion of ALDH^+^ cells in MCF-7 cells (Supplementary Fig. 1M). In addition, we isolated ALDH^-^ non-CSCs and ALDH^+^ CSCs from MDA-MB-231 cells by FACS (Fig. 1I). ALDH^-^ sorted cells spontaneously recovered original ALDH^+^ subpopulation after 6 days, while depletion of PYCR1 attenuated this transition (Fig. 1J, Supplementary Fig. 1N). Depletion of PYCR1 also suppressed the mammosphere-forming efficacy of ALDH^+^ sorted cells (Fig. 1K). These results uncover that PYCR1 exhibits essential roles in both resident CSC homeostasis and non-CSCs plasticity.

In vivo, NOD/SCID mice inoculated with shPYCR1 cells evidently formed smaller tumor masses than the mice injected with shNC cells (Fig. 1L, M), indicating that PYCR1 is essential for tumor growth. Meanwhile, the proportion of ALDH^+^ populations and the levels of stemness factors were significantly reduced after PYCR1 depletion (Supplementary Fig. 2A–D). We harvested primary tumor cells and performed reimplantation assay in serial dilutions in recipient mice. The results showed that tumor formation rates of PYCR1-deficient group were significantly decreased (Fig. 1N). Similar results were also found in PY8119 tumors (Supplementary Fig. 2E–I). Furthermore, NOD/SCID mice inoculated with spheroid cells showed that ablation of PYCR1 also inhibited tumor growth (Fig. 1O, P). Meanwhile, spheroid cells significantly accelerated tumor growth compared to monolayer culture cells (Fig. 1L, M, O, P). Collectively, these data establish that PYCR1 promotes cancer stem-like traits and breast malignancy.

### Proline mediates PYCR1-enhanced breast cancer stemness and tumor growth

**Fig. 2:**
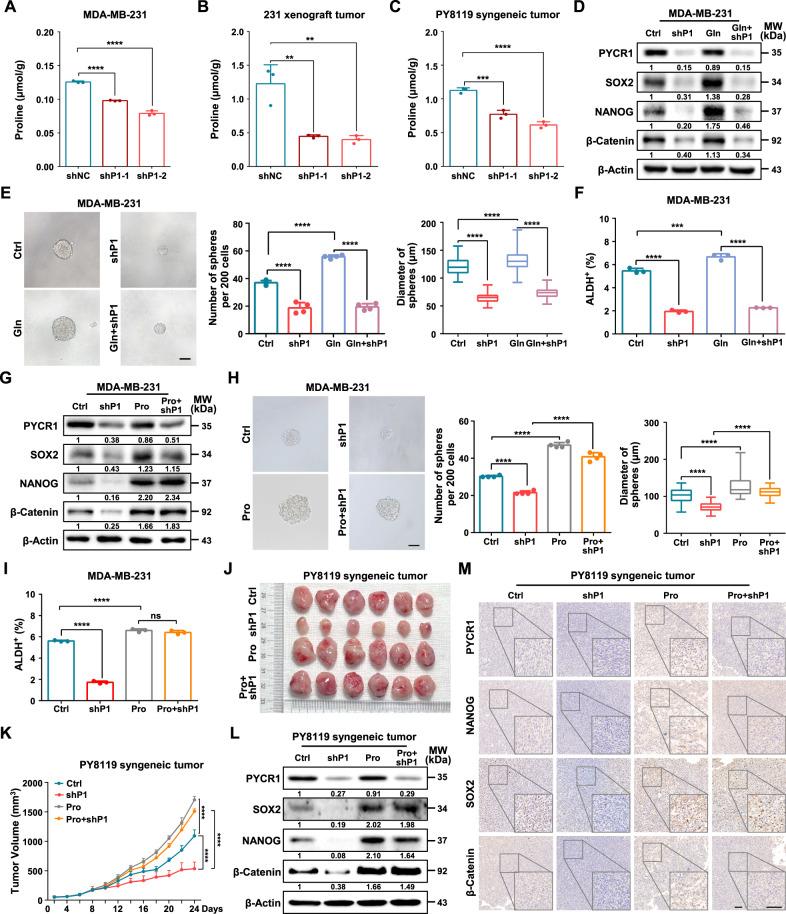
Proline mediates PYCR1-enhanced breast cancer stemness and tumor growth. **A**–**C** The proline level in MDA-MB-231 cells (**A**), MDA-MB-231 tumor (**B**) and PY8119 tumor (**C**) between shNC and PYCR1-silencing group (*n* = 3). **D** Relative protein levels of PYCR1, SOX2, NANOG and β-Catenin were determined following PYCR1 knockdown and supplement of glutamine (Gln) in MDA-MB-231 cells. **E** Sphere formation ability was analyzed following depletion of PYCR1 and supplement of glutamine in MDA-MB-231 cells. The representative images were presented (Left, scale bar = 100 μm) and the number (Middle) and diameter (Right) of spheroids was measured and counted (*n* = 4). **F** ALDH-positive cells in MDA-MB-231 cells following knockdown PYCR1 and supplement of glutamine were analyzed (*n* = 3). **G** Relative protein expression of PYCR1, SOX2, NANOG and β-Catenin were determined following PYCR1 knockdown and supplement of proline (100 μM) in MDA-MB-231 cells. **H** Sphere formation ability was analyzed following depletion of PYCR1 and supplement of proline in MDA-MB-231 cells. The representative images were presented (Left, scale bar = 100 μm) and the number (Middle) and diameter (Right) of spheroids was measured and counted (*n* = 4). **I** ALDH-positive cells in MDA-MB-231 cells following knockdown PYCR1 and supplement of proline were analyzed (*n* = 3). **J**, **K** C57BL/6 J mice (*n* = 6) were subcutaneously inoculated with PY8119 cells with PYCR1 silencing and supplement of proline every day (**J**), and tumor volumes were monitored (**K**). **L** Relative protein expression of PYCR1, SOX2, NANOG and β-Catenin were determined in xenograft PY8119 tumors. **M** Representative images of PYCR1, NANOG, SOX2 and β-Catenin by IHC staining in PY8119 xenograft tumors. Scale bars, 50 μm. Gln, glutamine. Pro, proline. Graph data were presented as mean ± SD. **P < 0.01, ***P < 0.001, ****P < 0.0001. *P*-values were calculated with two-tailed, unpaired Student’s *t*-test (**A**, **B**, **C**, **K**) or one-way ANOVA (**E**, **F**, **H**, **I**).

As the canonical function of PYCR1 is to catalyze proline synthesis [33], we found that proline level was significantly reduced in PYCR1-deficient cells and tumors (Fig. 2A–C). We then examined the role of proline in the maintenance of cancer stemness by supplement of physiological concentrations of proline in vitro [34]. The results showed that stemness-related factors were highly expressed in the proline-treated cells (Supplementary Fig. 3A, B). In addition, both the number and diameter of spheroids derived from the proline-treated cells were significantly increased compared to those from control cells (Supplementary Fig. 3C). Proline also elevated the proportion of ALDH^+^ populations in breast cancer cells (Supplementary Fig. 3D). Moreover, proline promoted the transition from ALDH^-^ non-CSCs to ALDH^+^ CSCs after sorting 3 days (Supplementary Fig. 3E) and enhanced the mammosphere-forming capacity of sorted ALDH^+^ cells (Supplementary Fig. 3F). In addition, proline promoted wound-healing and invasion of MDA-MB-231 cells (Supplementary Fig. 3G, H).

We next determine the dependent-effect of PYCR1 depletion on stemness function. As glutamine is a precursor for the synthesis of P5C, which is converted into proline by PYCR1 in mitochondria [35]. We therefore treated glutamine in PYCR1-deficient cells and detected the stem-like traits. The results showed that supplement of glutamine had no effect on proline levels (Supplementary Fig. 4A), the protein and mRNA expression levels of stemness factors (Fig. 2D, Supplementary Fig. 4B), sphere formation ability (Fig. 2E) and ALDH^+^ subpopulations (Fig. 2F, Supplementary Fig. 4C) in PYCR1-deficient cells. We further identified whether proline mediates PYCR1-induced stem-like properties and cancer progression. Supplement of proline rescued the mRNA and protein expression of stemness-related factors (Fig. 2G, Supplementary Fig. 4D), sphere formation capacity (Fig. 2H) and ALDH^+^ subpopulations (Fig. 2I, Supplementary Fig. 4E) in PYCR1-silencing cells. In addition, mice daily treated with physiological levels of proline [36] dramatically restored silencing of PYCR1-inhibited tumor growth (Fig. 2J, K). In syngeneic tumors, supplement of proline also rescued the PYCR1 knockdown-mediated decline in the protein and mRNA expression of stemness-related factors (Fig. 2L, M, Supplementary Fig. 4F).

We further verified whether proliferation and apoptosis levels of ALDH^-^ non-CSCs were affected by proline supplementation or PYCR1 depletion. The results showed that knockdown of PYCR1 attenuated the colony formation ability and cell viability, which could be reversed by proline (Supplementary Fig. 4G, H). In addition, ablation of PYCR1 induced apoptosis, which could be blocked by proline (Supplementary Fig. 4I). Altogether, these data demonstrate that proline mediates PYCR1-induced CSC maintenance and tumor development in TNBC.

### Proline activates cGMP-PKG signaling to promote breast cancer stem-like traits

**Fig. 3:**
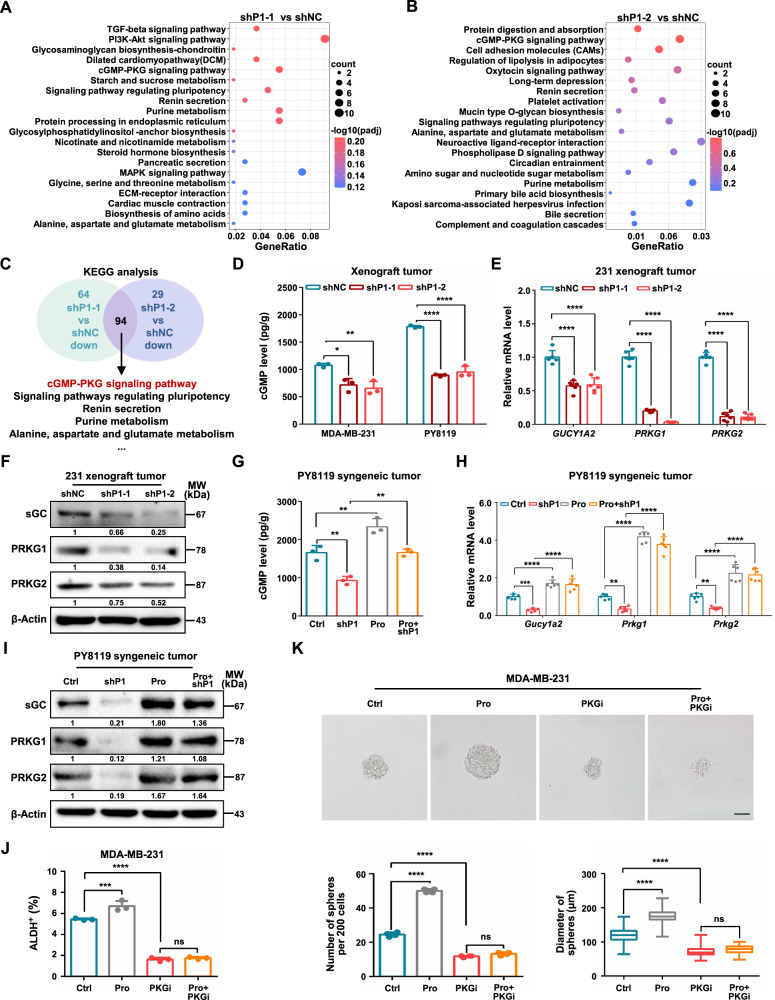
Proline activates cGMP-PKG signaling to promote breast cancer stem-like traits. **A**, **B** The enriched KEGG pathways of downregulated genes following shP1-1 (**A**) and shP1-2 MDA-MB-231 cells (**B**). **C** Overlapping the enriched KEGG pathways from downregulated genes of MDA-MB-231 shP1-1 cells and shP1-2 cells. The common pathways were listed. **D** The cGMP levels were measured in MDA-MB-231 tumor and PY8119 tumor following PYCR1 knockdown (*n* = 3). **E** Relative mRNA levels of *GUCY1A2*, *PRKG1* and *PRKG2* were determined following PYCR1 knockdown in MDA-MB-231 tumor (*n* = 6). **F** Relative protein levels of sGC, PRKG1 and PRKG2 were determined following PYCR1 knockdown in MDA-MB-231 tumor. **G** The cGMP levels were determined following PYCR1 knockdown and supplemental addition of proline in PY8119 tumor (*n* = 3). **H** Relative mRNA levels of *Gucy1a2*, *Prkg1* and *Prkg2* were determined following PYCR1 knockdown and supplemental addition of proline in PY8119 tumor (*n* = 6). **I** The protein levels of sGC, PRKG1 and PRKG2 were determined following PYCR1 knockdown and supplemental addition of proline in PY8119 tumor. **J** ALDH-positive populations were analyzed following treatment proline and PKGi in MDA-MB-231 cells. Differences of ALDH-positive cells among groups were analyzed (*n* = 3). **K** Sphere formation ability was analyzed following treatment proline and PKGi in MDA-MB-231 cells. The representative images were presented (Upper, scale bar = 100 μm) and the number (Bottom Left) and diameter (Bottom Right) of spheroids were measured and counted (*n* = 4). PKGi, PKG inhibitor. Graph data were presented as mean ± SD. *P < 0.05, **P < 0.01, ***P < 0.001, ****P < 0.0001. *P*-values were calculated with two-tailed, unpaired Student’s *t*-test (**D**, **E**) or one-way ANOVA (**G**, **H**, **J**, **K**).

To explore further the downstream targets responsible for the increase in proline-mediated stem-like traits in breast cancer, we performed RNA-seq based transcriptome analysis of PYCR1-knockdown (shP1-1 and shP1-2) versus control (shNC) cells. Among the differentially expressed genes (DEGs) identified in shP1-1 and shP1-2 cells, 361 genes were upregulated and 214 genes downregulated, respectively (Supplementary Fig. 5A, B). We then performed KEGG pathways enrichment analysis on the downregulated genes (Fig. 3A, B) and found that the cGMP-PKG signaling pathway was among the top 10 enriched pathways in both shP1-1 and shP1-2 groups (Fig. 3C). We then measured cGMP levels and found that depletion of PYCR1 significantly reduced cGMP in both breast tumors and cancer cells (Fig. 3D, Supplementary Fig. 5C). Consistently, ablation of PYCR1 decreased the mRNA and protein expression levels of all major cGMP-PKG signaling components, including GUCY1A2 (sGC), PRKG1 and PRKG2 (Fig. 3E, F and Supplementary Fig. 5D–G). Furthermore, intraperitoneal injections of proline into tumor-bearing mice rescued PYCR1 deficiency-induced decreased levels of cGMP and cGMP-PKG signaling related components in xenograft tumors (Fig. 3G–I). These results suggest that the cGMP-dependent pathway is a key downstream signaling pathway that promotes stemness mediated by proline.

Next, we measured the role of enzymatic activation of cGMP-dependent kinase PKG in proline-induced stem-like properties. We observed that PKG was highly activated in proline-treated cells, as revealed by the phosphorylation of its downstream factor vasodilator-stimulated phosphoprotein (p-VASP), which could be abolished by the PKG inhibitor (PKGi) KT5823 (Supplementary Fig. 5H). In concordance, the mRNA and protein levels of stemness-related factors were reduced in PKGi-treated cells (Supplementary Fig. 5I, J). We also found that inhibition of PKG kinase activity substantially reversed the proline-elevated ALDH^+^ subpopulations (Fig. 3J, Supplementary Fig. 5K) and inhibited the proline-enhanced sphere formation capacity (Fig. 3K). These experiments confirm a crucial role for cGMP-PKG signaling pathway in mediating proline-induced stem-like phenotypes in breast cancer.

### cGMP-PKG signaling mediates psychological stress-induced stem-like phenotypes and tumor development

**Fig. 4:**
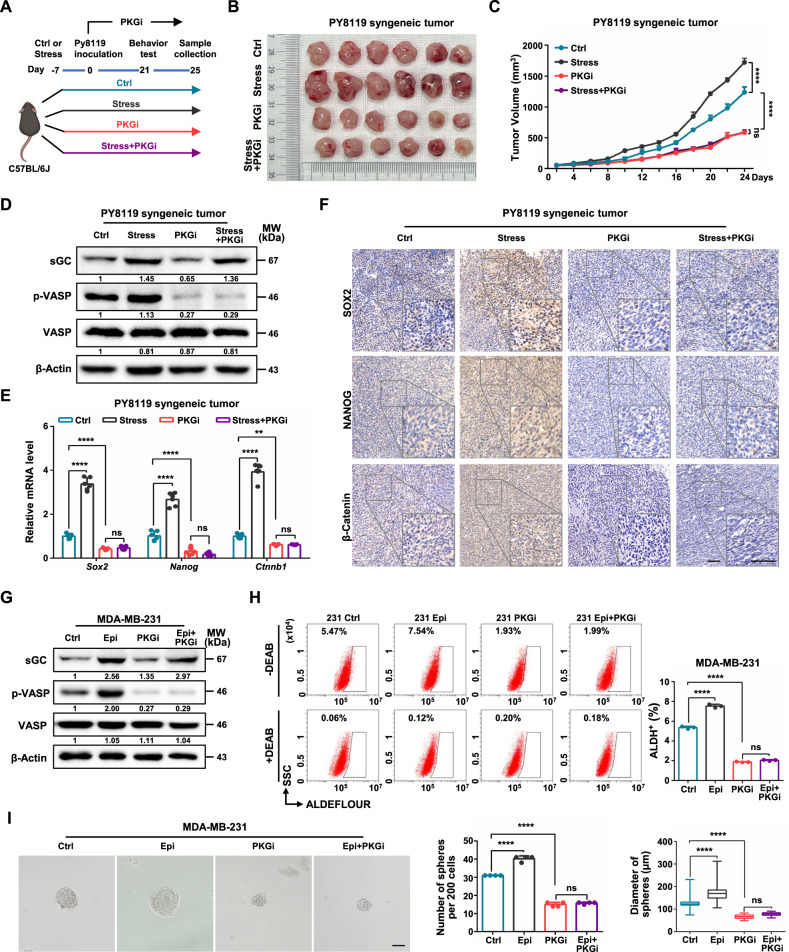
cGMP-PKG signaling mediates psychological stress-induced stem-like phenotypes and tumor development. **A** A schematic of the timing of chronic restraint stress, tumor inoculation, PKGi injections, behavioral tests and sample collection in the PY8119 syngeneic tumor model. **B**, **C** Representative tumor image (**B**) and growth curve (**C**) of Ctrl, Stress, PKGi and Stress plus PKGi PY8119 tumor in mice (*n* = 6). **D** Relative protein levels of sGC and phosphorylation level of VASP (Ser239) were determined following in PY8119 tumor (in A). **E** Relative mRNA levels of *Sox2*, *Nanog* and *Ctnnb1* were determined following in PY8119 tumor (*n* = 6). **F** Representative images of SOX2, NANOG and β-Catenin IHC staining in PY8119 tumor. Scale bars, 50 μm. **G** Relative protein levels of sGC and phosphorylation level of VASP (Ser239) were determined following Epi and PKGi treatment in MDA-MB-231 cells. **H** ALDH-positive populations were analyzed following treatment Epi and PKGi in MDA-MB-231 cells (*n* = 3). **I** Sphere formation ability was analyzed following treatment Epi and PKGi in MDA-MB-231 cells. The representative images were presented (Left, scale bar = 100 μm) and the number (Middle) and diameter (Right) of spheroids were measured and counted (*n* = 4). Epi, epinephrine. Graph data were presented as mean ± SD. **P < 0.01, ****P < 0.0001. *P*-values were calculated with two-tailed, unpaired Student’s *t*-test (**C**) or one-way ANOVA (**E**, **H**, **I**).

Breast cancer patients suffer from psychological stress, which promotes the CSC maintenance [28]. As such, psychological stress could impair spatial learning and memory in the hippocampus via disruption of cGMP-PKG signaling pathway [37]. Thus, we speculated that cGMP-PKG signaling pathway might mediates psychological stress-promoted cancer stemness. We took advantage of chronic restraint stress model [11, 38] in C57BL/6J mice, which were intraperitoneal injected with PKGi to inhibit cGMP-PKG signaling (Fig. 4A). Under psychological stress treatment, there was no difference in body weight among all mice groups (Supplementary Fig. 6A). Behavioral assays were performed in mice to assess the validity of psychological stress model. Psychological stress caused anxiety-like behaviors of female C57BL/6J mice, as established by a significant reduction in exploration of the central area of the open-field test (OFT) (Supplementary Fig. 6B), in exploration of the light compartment in the light–dark box test (LDT) (Supplementary Fig. 6C) and in entry times and residence time in the open arm of an elevated plus maze (EPM) (Supplementary Fig. 6D) as well as an increased immobility in tail suspension test (TST) (Supplementary Fig. 6E). Besides, intraperitoneal injection with PKGi into tumor-bearing mice had no changes on behavioral tests.

We next determined the effects of the PKGi on tumor growth, cGMP-PKG signaling and cancer stemness in xenograft mouse model undergoing psychological stress. Following injection with the PKGi, tumor volumes in stressed-mice were significantly decreased as compared with those from the stress group (Fig. 4B, C). Moreover, PKGi reversed stress-elevated cGMP levels (Supplementary Fig. 6F) and the expression of cGMP-PKG signaling components (Fig. 4D and Supplementary Fig. 6G). Furthermore, administration of the PKGi reduced the stress-increased mRNA and protein levels of stemness-related factors (Fig. 4E, F). Our previous study showed that stress-related hormone epinephrine is critical for promoting psychological stress-enhanced breast cancer stem-like properties [11]. Here, our results showed that the PKGi also reversed the increase in levels of both cGMP-PKG related components and cancer stemness-related factors caused by epinephrine (Fig. 4G, Supplementary Fig. 6H). In addition, the PKGi substantially diminished the ALDH^+^ subpopulations (Fig. 4H) and sphere formation ability (Fig. 4I) in epinephrine-treated cells. Collectively, these findings illustrate that cGMP-PKG signaling has an essential role in psychological stress-induced breast cancer stem-like traits.

### Silencing of PYCR1 reverses psychological stress-induced proline synthesis, cGMP-PKG signaling and cancer progression

**Fig. 5:**
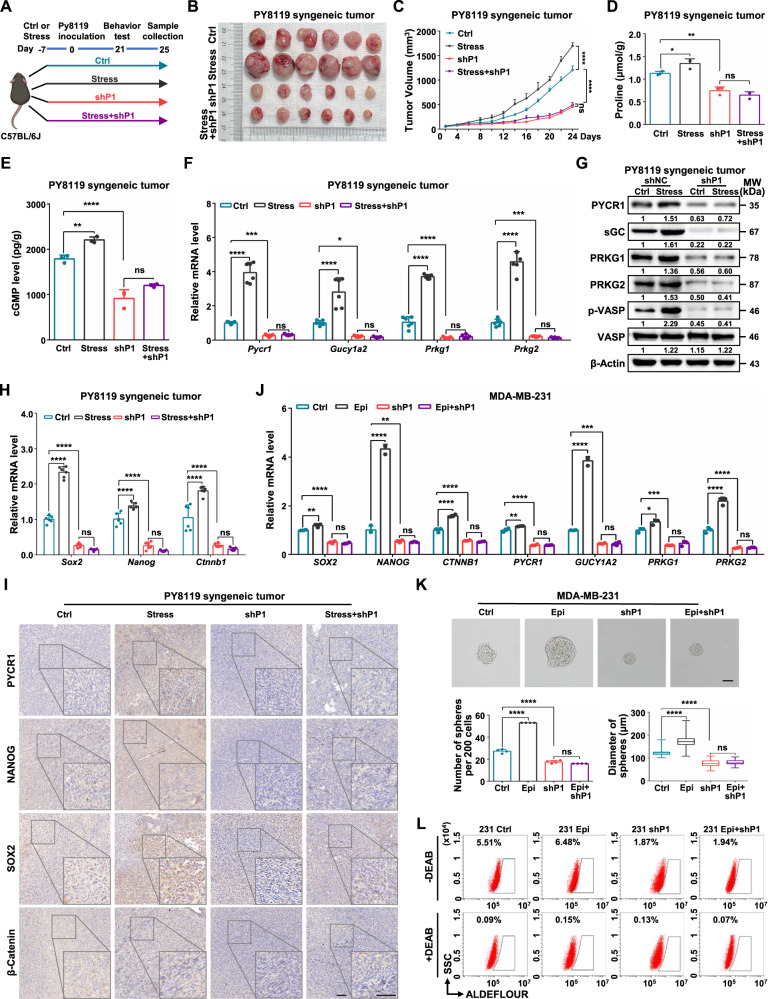
Silencing of PYCR1 reverses psychological stress-induced proline synthesis, cGMP-PKG signaling, and cancer progression. **A** A schematic of the timing of chronic restraint stress, tumor inoculation, behavioral tests and sample collection in the PY8119 syngeneic tumor model. **B**, **C** Representative tumor image (**B**) and growth curve (**C**) of Ctrl, Stress, shP1, and stress-induced shP1 PY8119 tumor in mice. **D** The proline levels were measured in PY8119 tumors (*n* = 3). **E** The cGMP levels were measured in PY8119 tumor (*n* = 3). **F** Relative mRNA levels of *Pycr1*, *Gucy1a2*, *Prkg1* and *Prkg2* were determined in PY8119 tumor (*n* = 6). **G** Relative protein levels of PYCR1, cGMP-PKG related genes and phosphorylation level of VASP (Ser239) were determined in PY8119 tumor. **H** Relative mRNA levels of *Sox2*, *Nanog* and *Ctnnb1* were determined in PY8119 tumor (*n* = 6). **I** Representative images of PYCR1, NANOG, SOX2 and β-Catenin IHC staining in PY8119 tumor. Scale bars, 50 μm. **J** Relative mRNA levels of *PYCR1*, stemness-related factors and cGMP-PKG signaling components were determined following PYCR1 knockdown and Epi treatment in MDA-MB-231 cells (*n* = 3). **K** Sphere formation ability was analyzed following PYCR1 knockdown and Epi treatment in MDA-MB-231 cells (*n* = 4). The representative images were presented (Upper, scale bar = 100 μm) and the number (Bottom Left) and diameter (Bottom Right) of spheroids were measured and counted. **L** Flow cytometry analysis for ALDH-positive cells in MDA-MB-231 cells following PYCR1 depletion and Epi treatment. Graph data were presented as mean ± SD. *P < 0.05, **P < 0.01, ***P < 0.001, ****P < 0.0001. *P*-values were calculated with two-tailed, unpaired Student’s *t*-test (**C**) or one-way ANOVA (**D**, **E**, **F**, **H**, **J**, **K**).

To investigate further whether the PYCR1-synthesized proline mediated psychological stress-induced tumor growth, cGMP-PKG signaling and cancer stemness, PYCR1-deficient cells inoculated mice were subjected to psychological stress (Fig. 5A). The results showed that suppression of proline biosynthesis by silencing PYCR1 significantly reversed psychological stress-induced tumor progression (Fig. 5B–D). Compared with control mice, cGMP levels in tumors from stressed-mice were elevated, which could be reversed by ablation of PYCR1 (Fig. 5E). In consistent, psychological stress increased the expression of cGMP-PKG mRNA and protein signaling components, whereas ablation of PYCR1 reversed these changes (Fig. 5F, G). Importantly, ablation of PYCR1 could significantly reverse stress-elevated stemness-related factors (Fig. 5H, I, Supplementary Fig. 7A).

In addition, we found that suppression of proline biosynthesis by ablation of PYCR1 also reversed epinephrine-upregulated cGMP-PKG signaling components and stemness-related factors (Fig. 5J), epinephrine-enhanced sphere formation ability (Fig. 5K) and epinephrine-increased ALDH^+^ populations (Fig. 5L, Supplementary Fig. 7B). Besides, depletion of PYCR1 also repressed the expression of β-adrenergic receptors and G protein in breast cancer cells (Supplementary Fig. 7C, D), which were the canonical downstream pathway components of psychological stress [28]. Moreover, ablation of PYCR1 significantly reverted the levels of stress-elevated β-adrenergic receptors and G proteins in tumors (Supplementary Fig. 7E). Collectively, these data demonstrate that suppression of proline biosynthesis by targeting PYCR1 reverses psychological stress-induced cGMP-PKG signaling and tumor development.

### Clinical relevance of PYCR1 and cGMP-PKG signaling in breast cancer patients

**Fig. 6:**
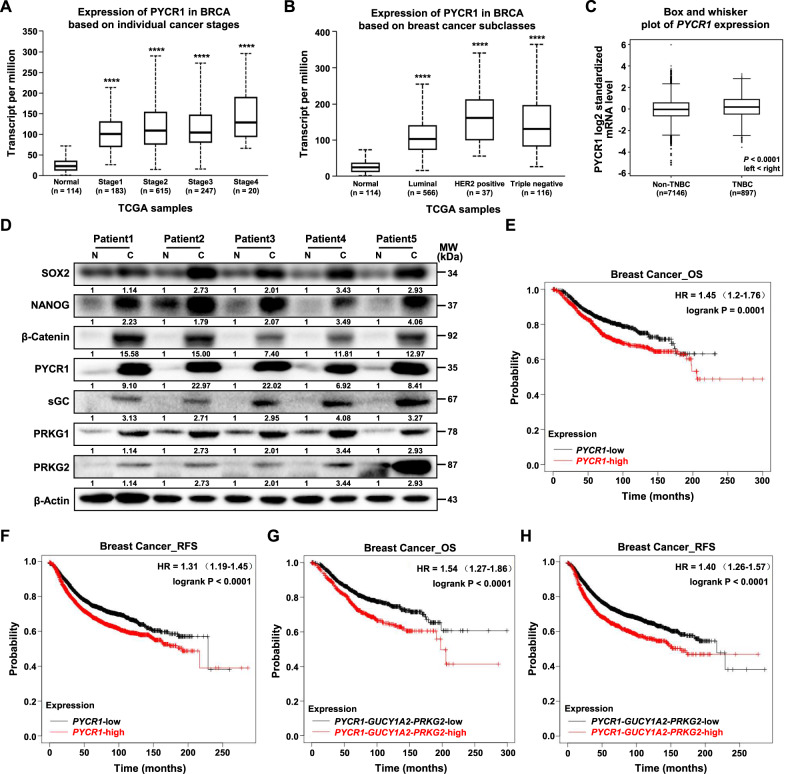
Clinical relevance of PYCR1 and cGMP-PKG signaling in breast cancer patients. **A**, **B** Box plot comparison of *PYCR1* expression in breast cancer based on individual cancer stages (**A**) and PAM50 breast cancer subtypes (**B**), ranked by median score within each subtype. These results are based upon data generated by the UALCAN Network (http://ualcan.path.uab.edu/). **C** Box and whisker plot comparison of *PYCR1* expression according to TNBC status created using database bc-GenExMiner v4.8. **D** Immunoblot analysis of PYCR1, stemness-related factors and cGMP-PKG related components in adjacent normal tissues (**N**) and TNBC tumor tissues (**C**). **E**, **F** Kaplan–Meier overall survival (OS) (**E**) and relapse free survival (RFS) (**F**) plots of breast cancer patients created using Kaplan–Meier Plotter network. Patients were classified into *PYCR1* high and *PYCR1* low subgroups and analyzed as indicated. **G**, **H** Kaplan–Meier overall survival (**G**) and relapse free survival (**H**) plots of breast cancer patients created using Kaplan–Meier Plotter network. Patients were classified into *PYCR1-GUCY1A2-PRKG2* high and *PYCR1-GUCY1A2-PRKG2* low subgroups and analyzed as indicated. ****P < 0.0001.

To evaluate the clinical relevance of PYCR1 in breast cancer patients, we validated the expression of PYCR1 and downstream cGMP-PKG components and predicted their prognosis using The Cancer Genome Atlas (TCGA) database. We found that high levels of PYCR1 were positively associated with advanced clinical stages of breast carcinomas (Fig. 6A). Compared with other breast cancer types, increased expression of PYCR1 was strongly associated with triple negative breast cancer status (Fig. 6B, C). We next collected 5 pairs of TNBC tumor tissues and adjacent normal tissues to perform Western blot analysis and the results showed that all TNBC tumor tissues displayed higher levels of PYCR1, cGMP-PKG signaling components and stemness-related factors compared with adjacent normal tissues (Fig. 6D). Importantly, breast cancer patients with elevated expression of PYCR1 and cGMP-PKG signatures exhibited significantly poor survival rates (Fig. 6E–H, Supplementary Fig. 8A–D). Altogether, these findings suggest that PYCR1-cGMP-PKG axis is a potential biomarker and a therapeutic target for breast cancer, especially TNBC.

## Discussion

In this study, we report the crucial role and the novel mechanism of proline metabolism regulated by PYCR1 in cancer stem-like cell maintenance, especially under psychological stress. Using both in vitro and in vivo established models, firstly we document that PYCR1-synthesized proline is required for breast cancer stem-like phenotypes. Secondly, we identify that proline activates cGMP-PKG signaling to enhance cancer stemness. Furthermore, we uncover that PYCR1/proline/cGMP-PKG axis mediates psychological stress-induced tumor growth and cancer stem-like traits. Finally, targeting proline metabolism or downstream cGMP-PKG signaling reverses psychological stress-induced breast cancer progression.

Proline modifies the ESC phenotypic and molecular characteristics towards mesenchymal-like and invasive pluripotent stem cell traits [39]. However, whether PYCR1-mediated proline metabolism regulates CSC maintenance remains unclear. In present study, our finding fills the void in knowledge by highlighting the role of PYCR1 in both triggering BCSC proliferation and accelerating transition from non-BCSCs into BCSCs through synthesizing proline. We further uncover that CSC-enriched spheroid cells present higher expression of another homologous PYCR isoform-PYCR2, which might also promote CSC traits. As the biosynthesis of proline occurs from glutamine in the mitochondria by PYCR1 and PYCR2 [39], high abundance of glutamine in TNBCs [40] could drive PYCR1-synthesized proline to promote BCSC maintenance. Indeed, our results ensure that inhibition of PYCR1 dramatically reverses glutamine-induced proline production and cancer stemness. Consistent with previous study in other cancer types [41, 42], our data show that silencing of PYCR1 decreases proline level to inhibit proliferation and induce apoptosis in TNBC cells, these phenotypes also contribute to PYCR1-mediated tumor growth. More intriguingly, the tumor growth inhibited by PYCR1-deficiency in immunocompetent mice was superior to that in immunodeficient mice. These findings imply that PYCR1-mediated proline metabolic reprogramming could affect anti-tumor immunity in TNBC, which is worth further investigating in the future study.

PYCR1-mediated proline metabolism regulates multiple cancer phenotypes [43], whereas the mechanism by which PYCR1 underlines cancer stemness has hitherto remained unknown. Using RNA-seq analysis in TNBCs following PYCR1 depletion, cGMP-PKG signaling pathway responsible for promoting malignancy in numerous cancer types [44–46], is identified as a novel downstream target of PYCR1. Further investigation confirms that PYCR1-synthesized proline activates cGMP-PKG signaling pathway to enhance cancer stemness. Although previous study has reported that GTP improves the amount of cGMP to activate PKG and the downstream MAPK pathway, contributing to breast cancer stem-like properties [47], how cGMP-PKG is activated by PYCR1 in BCSC maintenance remains unclear. As proline can be used to synthesize arginine [48] that in turn stimulates nitric oxide (NO)-soluble guanylate cyclase (sGC)-cGMP activation [49], this may explain the critical role of PYCR1-synthesized proline in cGMP-PKG signaling activation. In addition, we also find that PYCR1-synthesized proline elevates the mRNA and protein expression of sGC and PKG. But the transcriptional and post-transcriptional functions of proline are worthy of further investigation.

Psychological stress, closely associated with cancer incidence and mortality [50], releases stress-related hormones [28] to activate specific receptors that determine diverse tumoral biological processes, including proliferation, immune evasion and metabolic disorders [51]. For example, psychological stress-activated β2 adrenergic receptor triggers AR-cyclic AMP (cAMP)-protein kinase A (PKA) signaling to accelerate breast tumor growth [52]. As the cGMP pathway also functions directly downstream of the β2 receptor [53], cGMP-PKG could be involved in psychological stress-mediated oncogenic signaling. Indeed, our results show that blockage of PKG kinase activity effectively reverses psychological stress-induced tumor growth in vivo and counteracts epinephrine-enhanced cancer stem-like traits in vitro. However, little is known about whether PYCR1 mediates psychological stress-activated cGMP-PKG signaling in BCSC maintenance. In current study, psychological stress elevates mRNA and protein expression of PYCR1 and increases proline levels during tumor development. Silencing of PYCR1 effectively reverses psychological stress-induced cGMP-PKG activation and tumor growth, and further inhibits epinephrine-induced cancer stemness. To explain the potential mechanisms by which psychological stress increases PYCR1 expression, we speculate that (1) psychological stress-released epinephrine elevate the expression of MYC [11], a key transcriptional factor of PYCR1 [20] and (2) stress also elevate the expression of proinflammatory genes MZF1 [54], which acts as a transcriptional regulator to promote the expression of PYCR1 [55].

PYCR1 expression and proline metabolism are upregulated in various cancers [56], especially in invasive and poorly differentiated breast carcinoma [26, 57]. In concordance, we find that PYCR1, highly expressed in TNBC tumors and BCSCs, is correlated with poor survival and a higher risk of metastasis and recurrence in breast cancer patients. Hence, the tumoral proline level and PYCR1 expression could serve as potential biomarkers for TNBC diagnosis, especially metastasis status. Furthermore, chemotherapy significantly improves survival in early-stage breast cancer patients with low PYCR1 [25]. As PYCR1 can be inhibited by the small molecule inhibitor pargyline, combination therapy of pargyline with bortezomib ameliorated multiple myeloma tumor growth in murine model [42]. Therefore, PYCR1 inhibitor could act as a potential strategy to improve the chemotherapy efficacy for TNBC patients. In addition, increased intracellular cGMP-PKG signaling is closely associated with breast malignancy [58]. Inhibition of the NO/cGMP/PKG pathway reduces migration and invasion of human breast cancer cells [59]. In this study, blockage of the cGMP-PKG signaling pathway by PKG inhibitor dramatically reverses stress-induced tumor growth or proline-enhanced cancer stemness. Yet, these synergistic antitumor effects of PKG inhibitor provide a therapeutic opportunity to overcome drug resistance and metastasis for TNBC patients especially undergoing negative mood.

**Fig. 7:**
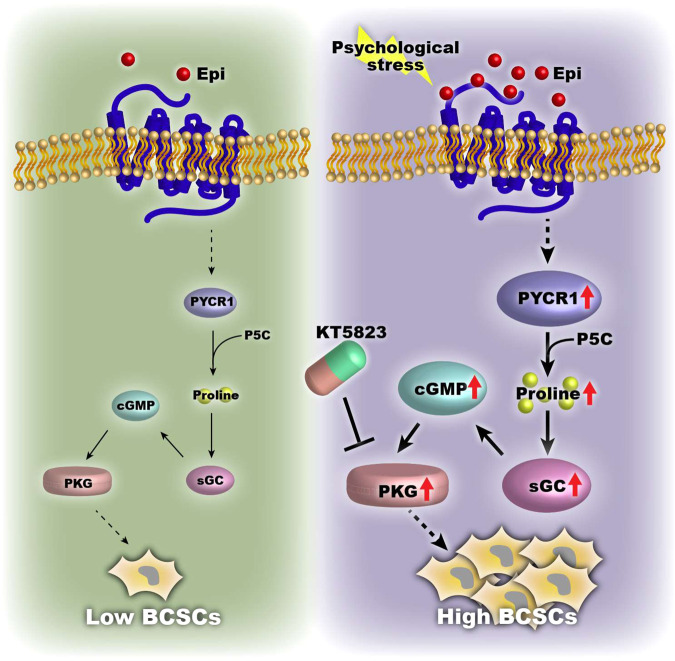
Working model of PYCR1-synthesized proline highlights breast cancer stemness under psychological stress. PYCR1-synthesized proline activates the cGMP-PKG signaling pathway to enhance TNBC stem-like properties under psychological stress. Targeting PYCR1-enhanced proline metabolic signaling provides a potential therapeutic approach for aggressive breast cancer patients undergoing psychological stress.

Altogether, our study reveals that PYCR1-synthesized proline activates the cGMP-PKG signaling pathway to enhance TNBC stem-like properties and that cGMP-PKG signaling mediates psychological stress-induced cancer stemness and progression. Targeting PYCR1-enhanced proline signaling provides a novel therapeutic approach for targeting aggressive breast cancer undergoing psychological stress (Fig. 7).

## Materials and methods

### Mice

C57BL/6J mice (4 weeks of age) were obtained from GemPharmatech and allowed to acclimatize to the animal facility for 2 weeks. Female NOD/SCID mice (4 weeks of age) were purchased from Charles River and adapted to the animal facility for 2 weeks. In all experiments, age- and gender-matched mice were used. Food and water were provided *ad libitum*. Prior to any interventions, mice were randomized to ensure that no incidental pre-intervention differences in body weight existed between the different groups.

### Breast cancer mouse models

Female NOD/SCID mice (6 weeks of age) were injected subcutaneously with the indicated number of MDA-MB-231 cells cultured in monolayer or sphere (1 × 10^6^ in PBS/Matrigel [1:1]). Murine breast cancer cells PY8119 (1 × 10^5^ in PBS/Matrigel [1:1]) were subcutaneously injected into both flanks of 6-week-old female C57BL/6J mice. Tumor size was measured using a vernier caliper and estimated using the formula = 0.5 × a × b^2^ (a and b were the long and short diameters of the tumors, respectively). For tissue collection, mice were anesthetized with isoflurane inhalation. All tumor-bearing mice were sacrificed at the ethical end point and tumors were immediately dissected and photographed.

For reimplantation assay in serial dilutions, primary xenografted tumors were digested with both collagenase I (Gibco, 17018029), and Dnase I (Merck, 10104159001). Serially diluted single cell suspensions (5000, 500, 50) were subcutaneously injected into NOD/SCID mice. After about 10 days (d), tumor formation ability was calculated using the Extreme Limiting Dilution Analysis website [60].

### Chronic restraint stress mouse model

As previously described [38], mice were placed in a perforated 50 ml conical tubes with holes to allow for air flow. They were maintained in the tubes for 6 h (10:00–16:00) per day for a maximum length of 30 days with the head facing the conical end of the tube to prevent them from moving freely or turning around without undue compression.

### Behavioral paradigms

Behavioral tests (*n* = 6) were performed on the day following the final restraint session in the dim light before initiation of behavioral tests, all mice were handled for at least 5 days for 5–10 min (min) per day to reduce the stress introduced by contact with the experimenter. Animals were habituated to the recording room for 90 min before testing. All the tests were completed on a single mouse within a couple of hours following the last restraint session, which in the order of Open field, Elevated plus maze, Light–dark box and Tail suspension. Movements of the animals were recorded by a video camera and analyzed using Xeye Aba (Beijing MacroAmbition S&T Development Co., Ltd). Test instruments were cleaned with 75% ethanol before each new test in order to remove any remaining odor, urine, and feces.

### Open field

An open field chamber made of acrylic (50 × 50 × 35 cm) was divided into a central field (center, 25 × 25 cm) and an outer field. Individual mice were placed in one corner of the chamber and their behavior was monitored with an overhead video camera for 5 min, including total crossing distance, times of center entries, time spent in the center and crossing distance in the center.

### Light-dark box

This apparatus consisted of a light box (50 × 50 × 35 cm) and dark box (50 × 50 × 35 cm), with the two boxes connected by an open door (50 × 50 × 35 cm). Mice were placed in the center of light box and with its back to the open door. The video cameras were embedded in the top of the boxes to monitor their behavior for 5 min, including numbers of entries into the light, time spent in both the light and dark boxes.

### Elevated plus maze

The maze apparatus, elevated to a height of 100 cm above the floor, consisted of two open arms (27 cm × 5.5 cm) and two closed arms (27 cm × 5.5 cm) that extended from a central platform (5.5 cm × 5.5 cm). Individual mice were placed in the central platform and allowed to explore for 5 min. The numbers of entries and time spent in open arm were recorded.

### Tail suspension

Mice were suspended by their tails with adhesive tape, which prevented them from escaping or touching nearby surfaces. The amount of time the mice remained immobile was recorded for 6 min.

### Cell culture

Human breast cells MCF-10A, MCF-7, MDA-MB-231, SK-BR-3 and BT549 were purchased from the American Type Culture Collection (ATCC). The murine breast cancer PY8119 cell line was purchased from ATCC. MCF-10A cells were cultured in Mammary Epithelial Cell Growth Medium BulletKit (Lonza/Clonetics Corporation). MCF-7 cells were maintained in Minimum Essential Medium (Gibco) supplemented with 10% fetal bovine serum (FBS) (Gibco) and 10 μg/ml insulin (Sigma). MDA-MB-231 cells were cultured in Leibovitz’s L-15 Medium (Gibco) supplemented with 10% FBS. SK-BR-3 cells were cultured in McCoy’s 5a Medium (Gibco) supplemented with 10% FBS, whereas BT549 cells were maintained in RPMI-1640 (Gibco) containing 10% FBS. The PY8119 cell line was cultured in F12K (HyClone) supplemented with 5% FBS. HEK293T cells from ATCC were cultured in dulbecco’s modified eagle medium (Gibco) containing 10% FBS. Incubate the MDA-MB-231 cells at 37 °C incubator without CO_2_. Other cells were maintained at 37 °C, 5% CO_2_. Anti-mycoplasma reagent SaveIt (Hanbio) and 0.1% penicillinstreptomycin (Thermo) were used in all cell culture system. All cell lines were authenticated by STR profiling. Petri dishes and cell culture plates were purchased from Jet Bio-Filtration Co., Ltd (Guangzhou, China) and NEST Biotechnology Co. Ltd. (Wuxi, China).

### Lentivirus packaging and generation of stable cell lines

Lentivirus was packaged with the 2nd generation packaging system plasmids psPAX2 and pMD2.G. HEK293T cells were co-transfected with lentiviral plasmids, psPAX2 and pMD2.G using Lipo2000 (Invitrogen). Culture medium containing the generated lentiviruses was collected 48 and 72 h (h) after transfection, respectively, and stored at −80 °C as aliquots. For infection with the lentivirus, cells were infected and subsequently selected with puromycin (2 μg/ml, Sigma).

### Plasmid construction

The shRNA fragment targeting PYCR1 was inserted into pLKO vector. Plasmids encoding human and murine PYCR1 were generated by PCR amplification and then subcloned into pLKO expression vectors. Plasmids encoding human PYCR1 were generated by PCR amplification and subcloned into pLVX expression vectors. The fidelity of all vectors was confirmed by DNA sequencing. All the primers used for plasmid construction are listed in Supplementary Table 1.

### Pharmacological studies in mice and breast cancer cells

Mice were injected intraperitoneally (i.p.) with 0.8 mg/kg proline daily (MCE) or 1 mg/kg KT5823 (MCE) every other day. Sterile 0.9% NaCl solution was used as the vehicle control. In the subcutaneous xenograft model, treatment was initiated 2 days after tumor-cell inoculation and continued until tissue dissection. Cells were maintained in medium that was supplemented with 2% FBS for 12 h, 48 h or 5 days with different pharmaceutical treatments. The concentrations of all drugs were chosen based on physiological concentration or previous publications. L-proline (100 μM, 200 μM; 12 h) (MCE), KT5823 (1 μM; 12 h) (MCE) [61, 62], glutamine (4 mM; 48 h) (Solarbio) [63], epinephrine bitartrate (10 nM; 5 days) (Selleck) [64, 65]. All the chemicals used in pharmacological studies are listed in Supplementary Table 3.

### Quantitative reverse transcription PCR (RT-qPCR)

Total RNA was extracted using TRIzol reagent (Life Technologies) and RNA concentration determined using a Nanodrop Spectrophotometer (Thermo Scientific). These RNAs were converted into cDNAs using an EasyScript One-Step gDNA Removal cDNA Synthesis SuperMix (Transgen) and Evo M-MLV RT Kit with gDNA Clean for qPCR (ACCURATE BIOTECHNOLOGY, HUNAN). RT-qPCR was performed using a 2 × Universal SYBR Green Fast qPCR Mix (ABclonal) and ChamQTM Universal SYBR qPCR Master Mix (Vazyme). The housekeeping gene ACTB was used as an internal control to normalize RNA expression. Primers used in RT-qPCR are given in Supplementary Table 2.

### Western blotting

After several gentle disruptions on ice, cells were lysed in RIPA buffer (50 mM Tris, pH 7.5, 120 mM NaCl, 1% Triton X-100, 0.5% sodium deoxycholate, 0.1% SDS, 5 mM EDTA) with a protease inhibitor cocktail (MCE) or Phosphatase Inhibitor Cocktail (Bimake) for 30 min. Protein lysates were quantified using the Coomassie bright blue assay. Equal amounts of protein were loaded on gels and separated by SDS-PAGE and transferred to nitrocellulose membranes (Millipore, HATF00010) to be incubated with primary and secondary antibodies. Proteins were exposed by incubating the membranes with ECL kits (Thermo Fisher Scientific). They were then quantified with a ChemiDoc MP Imaging System (Bio-Rad). Quantification of protein levels was normalized with β-Actin using ImageJ software (Version 1.8.0). Antibodies used for western blotting are given in Supplementary Table 3.

### Immunohistochemistry and histopathological examination

Analysis of PYCR1, SOX2, NANOG, β-Catenin, PRKG1 and PRKG2 was performed using SPlink Detection Kits (ZSGB-BIO). All steps were performed in accordance with the manufacturer’s instructions. The immunohistochemistry staining images were scanned using Pannoramic MIDI (3Dhistech). Commercial Detection Kits used for immunohistochemistry staining are given in Supplementary Table 3.

### ALDH activity assay

The ALDEFLUOR assay was carried out according to the manufacturer’s guidelines (STEMCELL Technologies). Briefly, primary xenografted tumors were digested with both collagenase I (Gibco), and Dnase I (Merck) to single cell state before assay. Total 5 × 10^5^ cells were suspended in 1 ml ALDEFLUOR assay buffer. This was followed by addition of 5 µl of activated ALDEFLUOR reagent to the sample tube and adding 5 µl of the ALDEFLUOR DEAB reagent to the negative control tube. After mixing the sample tubes, 0.5 ml of the mixture was immediately transferred to the negative control tube. Both the sample and negative control tubes were incubated for 30 min at 37 °C. Cells were collected and analyzed with a flow cytometer (Beckman Coulter, CytoFLEX). Commercial ALDH activity assay kit used for ALDH staining are given in Supplementary Table 3.

### Fluorescence-Activated Cell Sorting (FACS)

The ALDH^-^ and ALDH^+^ subpopulations were separated after ALDH staining reaction as previously described [66, 67]. In brief, cells were incubated with activated ALDEFLUOR reagent (5 µl reagent per 5 × 10^5^ cells) for 30 min at 37 °C. Subsequently, cells were collected and sorted on a flow cell sorter (Sony Corporation, LE-SH800S, Japan) for subsequent culture.

### Sphere formation assay

Cells were re-suspended as single cells in DMEM/F12 medium (Gibco). Then 200 cells/well were added in 1 ml DMEM/F12 supplemented with 1% methylcellulose (R&D Systems), 20 ng/ml basic fibroblast growth factor (Peprotech), 20 μl/ml B-27 (Gibco) and 20 ng/ml epidermal growth factor (Sigma) and seeded to a 24 well ultra-low attachment surface plates (Corning, 3473). Each group was plated in 4 wells. After 7–10 days, all formed spheroids were quantified from images using an inverted microscope (Olympus, DP73).

For Extreme limiting dilution assay (ELDA), cells enriched from spheroids were seeded into 96-well ultralow attachment plates (Corning, 3474) with sphere medium at density of 25/10/5/3/2 and 1 cell per well. After 7 d, positive (sphere formation) well numbers in each group were uploaded and calculated in the ELDA website [60].

### Wound healing assay

Cells were treated with proline for 12 h and then plated in six-well plates and reached to 100% confluence. Subsequently, wounds were scratched onto the monolayer of cells with a sterile pipette tip and then incubated in serum-free medium. For each well, at least five pictures were taken microscopically at 0 h and 48 h after scratching. The percentage of wound healing was determined based on three measurements of the wound area.

### Transwell invasion assay

Cells were pre-treated with proline for 12 h. For the invasion assay, the transwell upper chamber (24-well insert, 8 μm, Corning Costar, China) was coated with 50 μl Matrigel (BD Bioscience) and then cells (5 × 10^4^) were plated onto the top of the coated chamber in serum-free medium. Medium supplemented with 10% FBS were used as an attractant in the lower chamber. After being incubated for 36 h, cells invaded through the membrane were fixed with 4% paraformaldehyle (Santa Cruz) and stained with 0.4% crystal violet (Shanghai Sangon Company, China). The stained cell images were captured by microscope (Olympus, Japan), and five random fields at 10 × magnification were counted using ImageJ software (Version 1.8.0).

### Colony formation assay

Sorted ALDH^-^ MDA-MB-231 cells were seeded into 60 mm plastic dish plates at a cell density of 1000 cells/dish and were allowed to grow for 10–14 days until clones were visible. PBS-washed cells were fixed with 4% paraformaldehyde and stained with 0.4% crystal violet (Shanghai Sangon Company, China). Three stained colonies images were counted by ImageJ.

### Cell viability assay

Cell viability was measured using a Cell Counting Kit-8 (CCK-8, Meilunbio, China) according to the manufacturer’s instructions. Sorted ALDH^-^ MDA-MB-231 cells were seeded at a density of 5000 cells/well in 96-well plates and incubated overnight. Then the cells were treated with 100 μM proline for 12 h. Subsequently, CCK-8 solution (10 μL) was added to each well and incubated for 40 min. The absorbance was measured at 450 nm using a spectrophotometer. Commercial detection reagents used for cell viability assay are given in Supplementary Table 3.

### Annexin V‑FITC/PI double staining

Annexin V-FITC detection kit (Abbkine, Wuhan, China) was used to detect apoptosis in our models. Sorted ALDH^-^ MDA-MB-231 cells were collected using EDTA-free trypsin and then resuspended with PBS. After that, cells were stained with 5 μL of Annexin V-FITC and 2 μL of PI for 15 min at room temperature in the dark according to the manufacturer’s instructions. Next, the stained cells were analyzed by flow cytometry (Beckman Coulter, CytoFLEX). Commercial detection kit used for Annexin V‑FITC/PI double staining are given in Supplementary Table 3.

### Proline assay

Proline was determined using a validated proline assay kit (Solarbio), with all samples being extracted according to the manufacturer’s protocol. Briefly, cells were lysed by sonication while tumor tissues were lysed using a grinder. Supernatants were shaken and extracted in boiling water for 10 min. After centrifugation for 10 min, room temperature, 1000 × *g*, supernatants were collected. Absorbance at 520 nm was used to determine proline level. Commercial detection kit used for proline assay are given in Supplementary Table 3.

### Enzyme-linked immunosorbent assay (ELISA)

ELISA assays to measure cGMP levels in tumor cells and tissues were assayed per manufacturer’s instructions. Briefly, for tumor cells, the cell suspension was collected after washing with pre-cooled PBS. Next, cells were sonicated on ice using an Ultrasonic Sample Processing System (LICHEN) set at 30% of maximum power to fully lysed. Remove the cell fragments, collect the supernatant for cGMP measurement. For tumor tissues, tumor pieces were been weighed and then homogenized in PBS (tissue weight (g) : PBS (mL) volume = 1: 9) with a glass homogenizer on ice. The homogenates were then centrifuged for 5 min at 5000 × *g* to get the supernatant for cGMP measurement. Commercial detection kit used for ELISA are given in Supplementary Table 3.

### RNA-seq

TRIzol reagent (Invitrogen) was used to extract total RNAs from three replicate samples of MDA-MB-231 cells (shNC, shPYCR1-1, shPYCR1-2) and submitted to Novogene (Beijing, China) Libraries were prepared according to the NEBNext UltraTM RNA Library Prep Kit for Illumina (NEB) following the manufacturer’s recommendations. All samples were sequenced using the illumina Hiseq platform with pair end 125 bp read length. The RNA-seq data were mapped to the reference genome hg19 using the software Hisat2 v2.0.5 that was based on the default parameters. Expected number of fragments per kilobase of transcript sequences per million base pairs sequenced (FPKM) estimated each gene expression level by feature counts (v1.5.0-p3). Differentially expressed genes (DEGs) were analyzed using the DEseq2 R package with an adjusted *P*-value < 0.05. A log2 fold change > = 1 was assigned as differentially expressed. Gene Ontology (GO) enrichment analysis of DEGs was implemented by using cluster Profiler R package software (3.8.1). These sequence data have been submitted to the GenBank databases under accession number GSE220931.

### Software

Hazard ratios for tumor cohorts of breast cancer were determined using the Kaplan–Meier (KM) plotter online tool set to employ the best cutoff analyses and multigene classifier (http://kmplot.com/) [68]. Overall survival (OS), recurrence free survival (RFS) and distant metastasis-free survival (DMFS) of breast cancer patients were analyzed using Kaplan–Meier survival plots.

### Statistical analyses

Each in vivo and in vitro experiment was performed in triplicate and repeated at least 3 times. Statistical analyses were performed using either SPSS software (version 16.0) or GraphPad Prism 6.0 (GraphPad Software Inc.). Differences between variables were compared by two-tailed Student’s *t*-test or by one-way ANOVA with corrections for multiple comparisons. Tumor growth curve data were analyzed at the ethical end point using a two-tailed, unpaired Student’s *t*-test. Data were expressed as mean ± SD. *P*-values <0.05 were considered statistically significant (*P < 0.05, **P < 0.01, ***P < 0.001, ****P < 0.0001).

### Supplementary information

Supplementary Figures and Tables

Original Data File

Checklist

## Data Availability

The analysis data were downloaded from the GEO database under the accession number SRP157974 for comparing TNBC tumor tissue with adjacent normal tissues. The DEGs for spheroids and monolayer MDA-MB-231 cells were downloaded from the GEO database under the accession number GSE116180. The data that support the findings of this study are available from the corresponding author upon reasonable request.
