# Supplementary material for: Pyrroline-5-carboxylate reductase 1 reprograms proline metabolism to drive breast cancer stemness under psychological stress
Source: Cell Death Dis. 2023 Oct 16;14(10):682. doi: 10.1038/s41419-023-06200-5 (PMC10579265; doi:10.1038/s41419-023-06200-5)
Supplement: Supplementary file 1 — Supplementary Figures and Tables [file 41419_2023_6200_MOESM1_ESM.pdf]

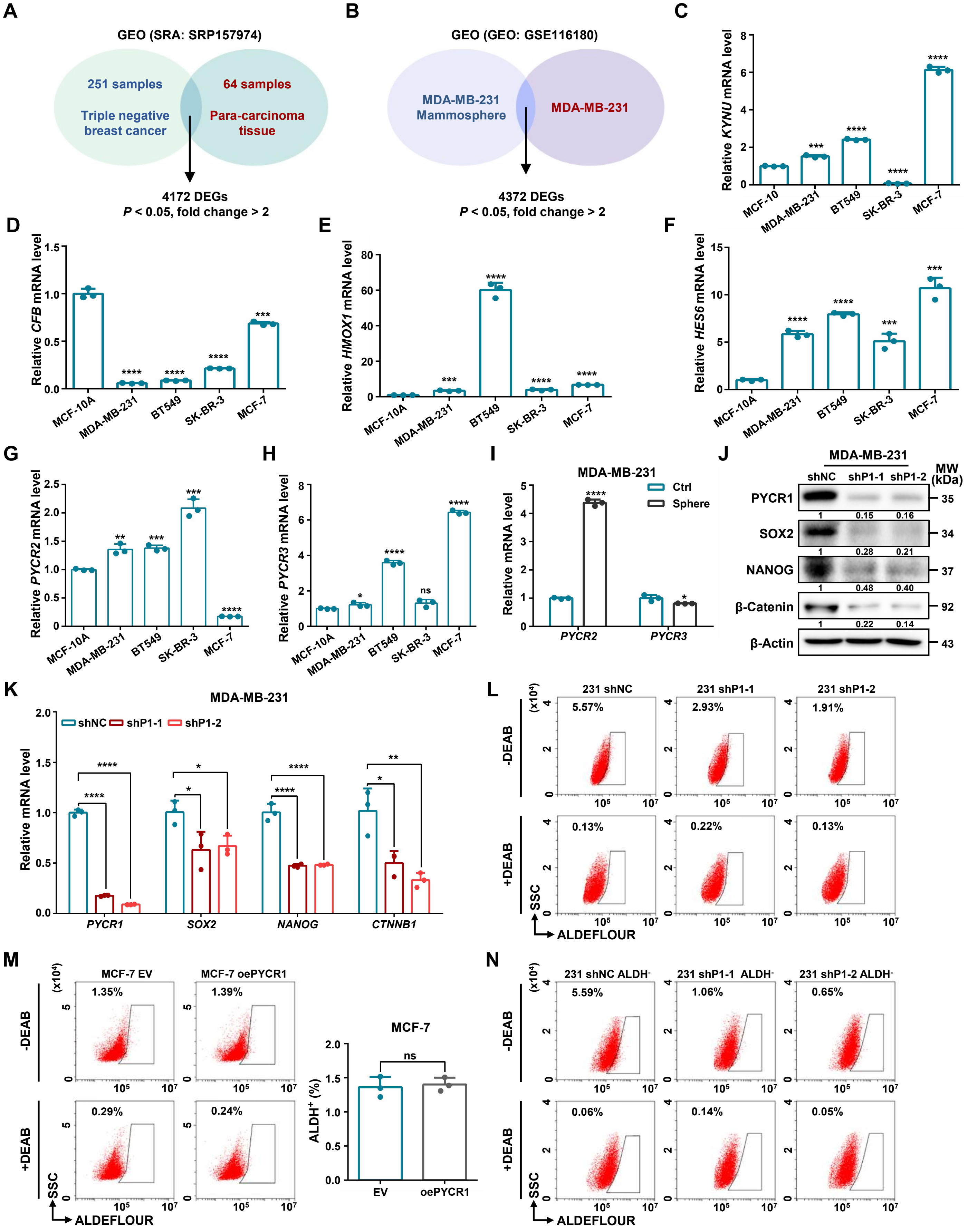

Supplementary Fig. 1

(**A**) Overlapping expressed genes from TNBC and normal breast tissues to obtain DEGs (SRP157974) (FC > 2, P < 0.05). (**B**) Overlapping expressed genes from spheroids and adherent MDA-MB-231 cells to obtain DEGs (GSE116180) (FC > 2, P < 0.05). (**C-H**) Relative mRNA levels of typical xenobiotic metabolism gene *KYNU* (**C**), *CFB* (**D**), *HMOX1* (**E**), *HES6* (**F**), *PYCR2* (**G**) and *PYCR3* (**H**) in breast cancer cell lines (n = 3). (**I**) Relative mRNA levels of *PYCR2* and *PYCR3* in sphere MDA-MB-231 cells (n = 3). (**J**) Relative protein levels of PYCR1, SOX2, NANOG and β-Catenin were determined in PYCR1 knockdown MDA-MB-231 cells. (**K**) Relative mRNA levels of *PYCR1*, *SOX2*, *NANOG* and *CTNNB1* were determined in PYCR1 knockdown MDA-MB-231 cells (n = 3). (**L**) Flow cytometry analysis for ALDH-positive cells in MDA-MB-231 cells following PYCR1 depletion. (**M**) Flow cytometry analysis for ALDH-positive cells in MCF-7 cells (Left). Differences of ALDH-positive cells were analyzed (Right, n = 3). (**N**) The ALDH-positive cells in sorted ALDH$^-$ MDA-MB-231 shNC, shP1-1, shP1-2 cells were detected by flow cytometry after 6 days monolayer culture. Graph data were presented as mean ± SD. *P < 0.05, **P < 0.01, ***P < 0.001, ****P < 0.0001. P values were calculated with two-tailed, unpaired Student's t-test.

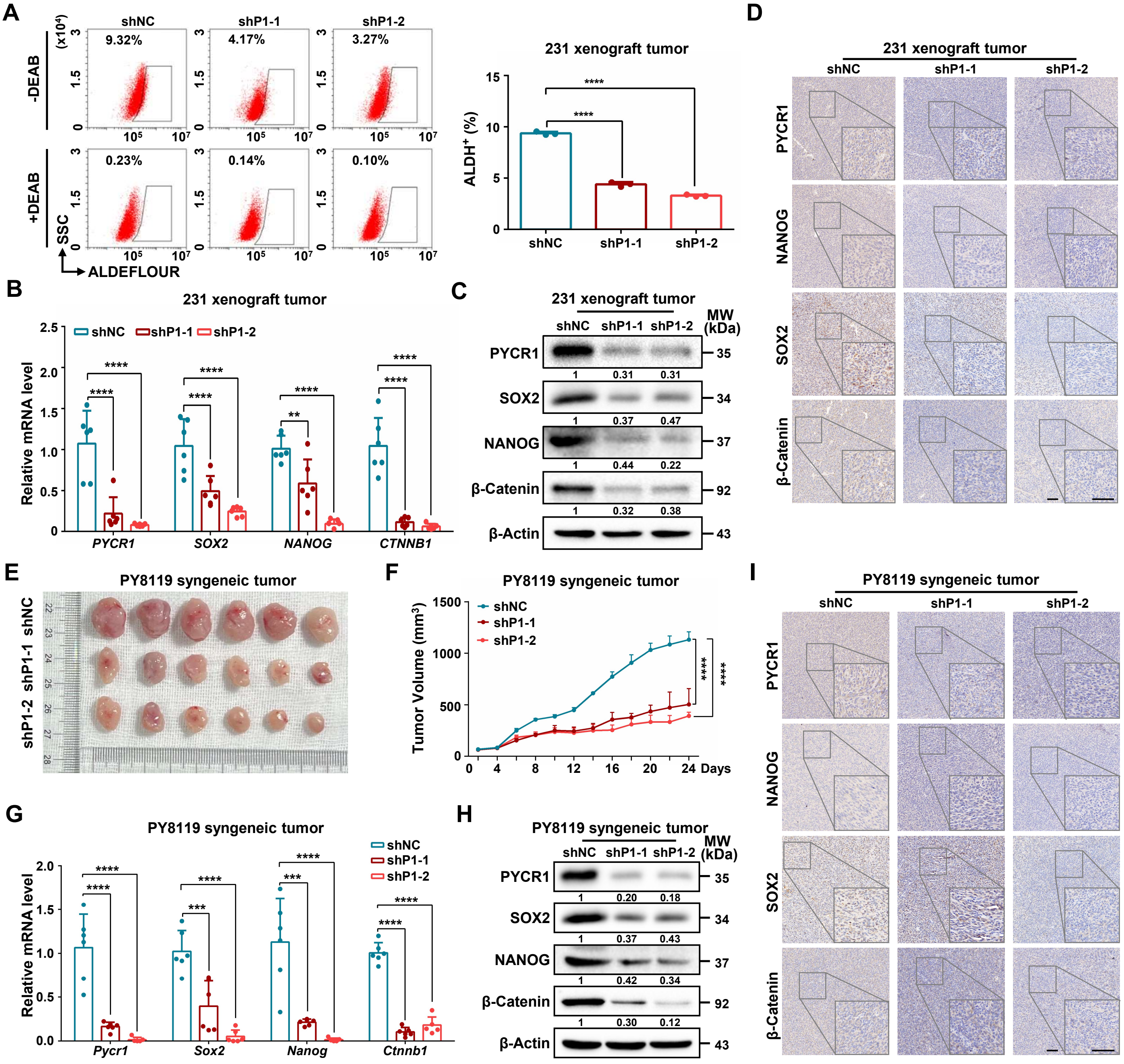

Supplementary Fig. 2

(**A**) ALDH-positive populations were analyzed in xenograft tumor (from Fig. 1L) (Left). Differences of ALDH-positive cells among groups were analyzed (n = 3, Right). (**B**) Relative mRNA levels of *PYCR1*, *SOX2*, *NANOG* and *CTNNB1* were determined between MDA-MB-231 shNC and shP1 (shP1-1, shP1-2) tumor (n = 6). (**C**) Relative protein levels of PYCR1, SOX2, NANOG and β-Catenin were determined between MDA-MB-231 shNC and shP1 (shP1-1, shP1-2) tumor. (**D**) Representative images of PYCR1, NANOG, SOX2 and β-Catenin IHC staining in MDA-MB-231 shNC and shPYCR1 (shP1-1, shP1-2) tumor. Scale bars, 50 μm. (**E-F**) Immunocompetent mice (n = 6) were subcutaneously inoculated with shPycr-1 (shP1-1) and shPycr-2 (shP1-2) PY8119 cells (**E**) and tumor volumes were analyzed (**F**). (**G**) Relative mRNA levels of *Pycr1*, *Sox2*, *Nanog* and *Ctnnb1* were determined between PY8119 shNC and shPycr1 (shP1-1, shP1-2) tumor (n = 6). (**H**) Relative protein levels of PYCR1, SOX2, NANOG and β-Catenin were determined between PY8119 shNC and shP1 (shP1-1, shP1-2) tumor. (**I**) Representative images of PYCR1, NANOG, SOX2 and β-Catenin IHC staining in tumor. Scale bars, 50 μm. Graph data were presented as mean ± SD. $^{**}P < 0.01$, $^{***}P < 0.001$, $^{****}P < 0.0001$. P values were calculated with two-tailed, unpaired Student's t-test.

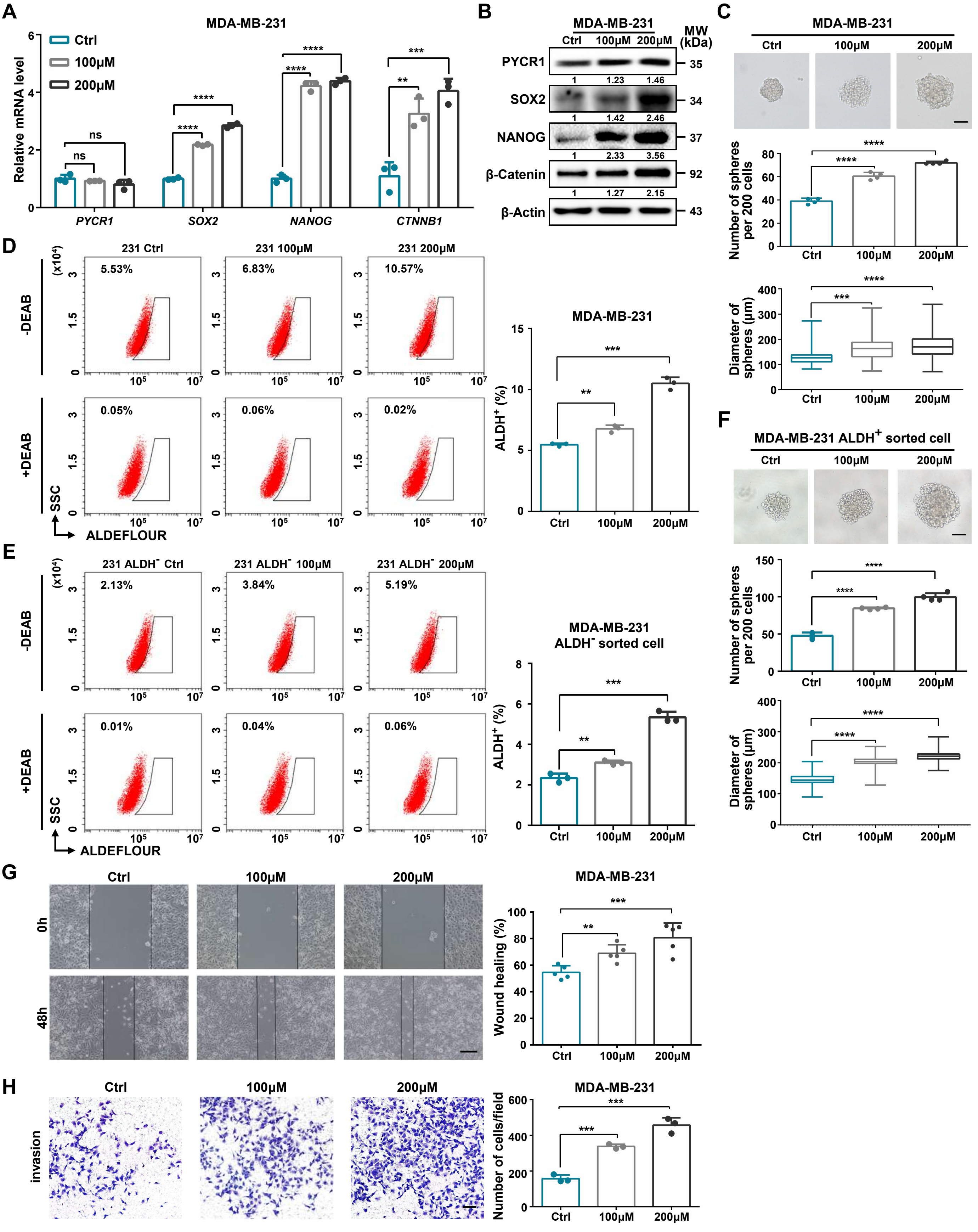

Supplementary Fig. 3

(**A**) Relative mRNA levels of *PYCR1*, *SOX2*, *NANOG* and *CTNNB1* were determined in MDA-MB-231 cells treated with proline (100 μM or 200 μM) (n = 3). (**B**) Relative protein levels of PYCR1, SOX2, NANOG and β-Catenin were determined in MDA-MB-231 cells treated with proline (100 μM or 200 μM). (**C**) Sphere formation was performed in MDA-MB-231 cells treated with proline (100 μM or 200 μM). The representative images were presented (Upper, scale bar = 100 μm) and the number (Middle) and diameter (Botton) of spheroids were measured and counted (n = 4). (**D**) ALDH-positive populations were analyzed following supplemental addition of proline to the MDA-MB-231 cells (Left). Differences of ALDH-positive cells among groups were analyzed (n = 3) (Right). (**E**) Flow cytometry analysis for ALDH-positive cells in sorted ALDH$^-$ MDA-MB-231 cells treated proline (100 μM or 200 μM) after 3 days monolayer culture. Differences of ALDH-positive cells among groups were analyzed (n = 3). (**F**) Sphere formation assay was performed in sorted ALDH$^+$ MDA-MB-231 cells treated proline (100 μM or 200 μM). The representative images were presented (Upper, scale bar = 100 μm) and the number (Middle) and diameter (Botton) of spheroids were measured and counted (n = 4). (**G**) Representative images of MDA-MB-231 cells treated proline (100 μM or 200 μM) in wound healing assay (Left). Statistical significance was analyzed (n =5) (Right, scale bar = 100 μm). (**H**) Representative images of MDA-MB-231 cells treated proline (100 μM or 200 μM) in transwell invasion assays (Left). Statistical significance was analyzed (n =3) (Right, scale bar = 100 μm). Graph data were presented as mean ± SD. $^{**}P < 0.01$, $^{***}P < 0.001$, $^{****}P < 0.0001$. *P* values were calculated with two-tailed, unpaired Student's t-test.

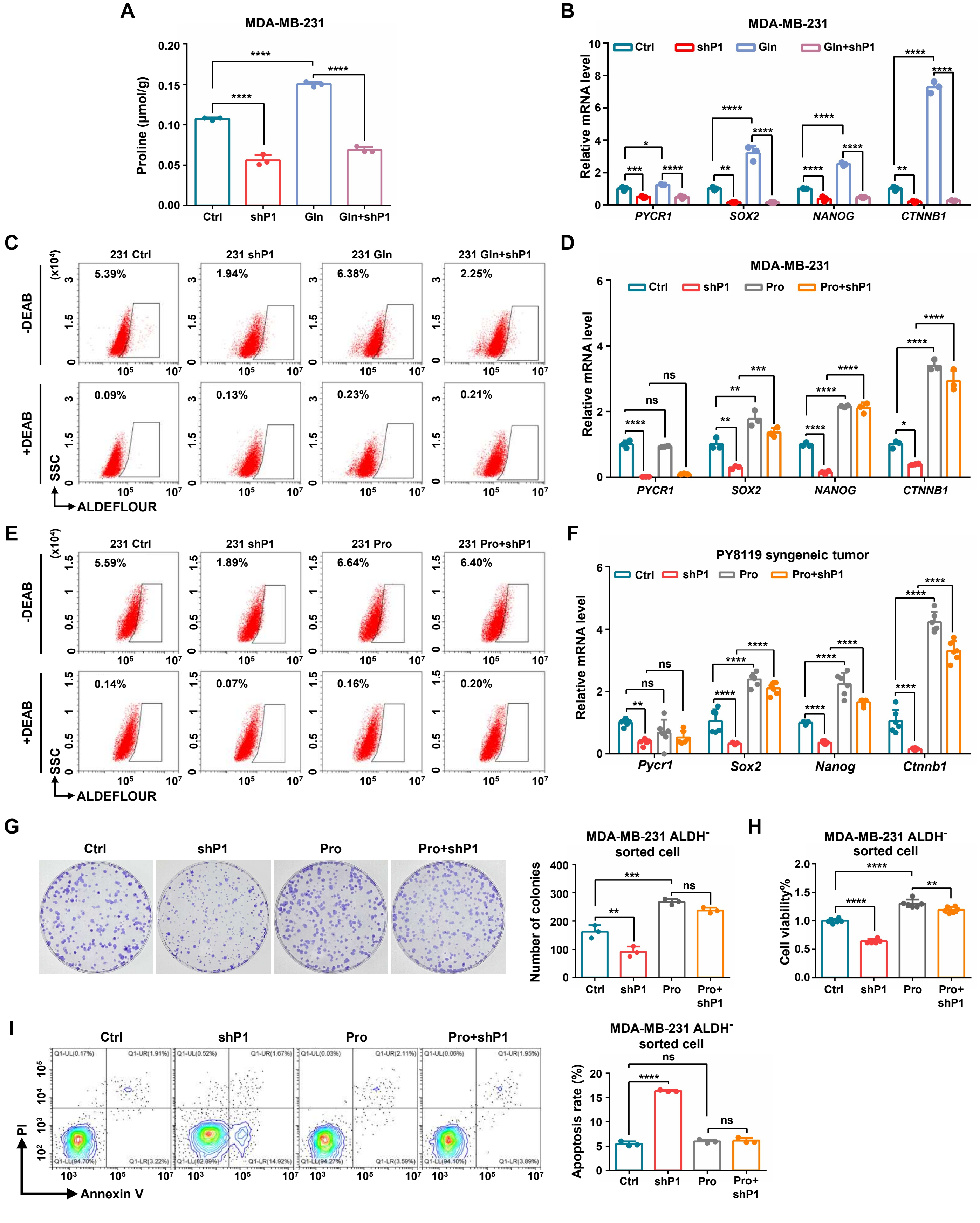

Supplementary Fig. 4

(**A**) The proline levels were measured in PYCR1-deficient MDA-MB-231 cells treated glutamine (n = 3). (**B**) Relative mRNA levels of *PYCR1*, *SOX2*, *NANOG* and *CTNNB1* were determined in PYCR1-deficient MDA-MB-231 cells treated with glutamine (n = 3). (**C**) Flow cytometry analysis for ALDH-positive cells in PYCR1-deficient MDA-MB-231 cells treated glutamine. (**D**) Relative mRNA levels of *PYCR1*, *SOX2*, *NANOG* and *CTNNB1* were determined in MDA-MB-231 cells treated with proline (n = 3). (**E**) Flow cytometry analysis for ALDH-positive cells in PYCR1-deficient MDA-MB-231 cells treated proline. (**F**) Relative mRNA levels of *Pycr1*, *Sox2*, *Nanog* and *Ctnnb1* were determined in PY8119 xenograft tumor (n = 6). (**G**) Representative images of colonies formed by sorted ALDH$^-$ MDA-MB-231 cells (Ctrl and shP1) following treated proline (Left). The numbers of colonies were analyzed (Right) (n = 3). (**H**) CCK-8 assay for sorted ALDH$^-$ MDA-MB-231 cells (Ctrl and shP1) following treated proline for 12 h. The cell viability was analyzed (n = 6). (**I**) Flow cytometry with Annexin V/PI double staining was used to detect the percentage of apoptosis in sorted ALDH$^-$ MDA-MB-231 cells (Ctrl and shP1) following treated proline (Left). Differences of apoptosis rate among groups were analyzed (n = 3) (Right). Graph data were presented as mean ± SD. *$P < 0.05$, **$P < 0.01$, ***$P < 0.001$, ****$P < 0.0001$. P values were calculated with one-way ANOVA.

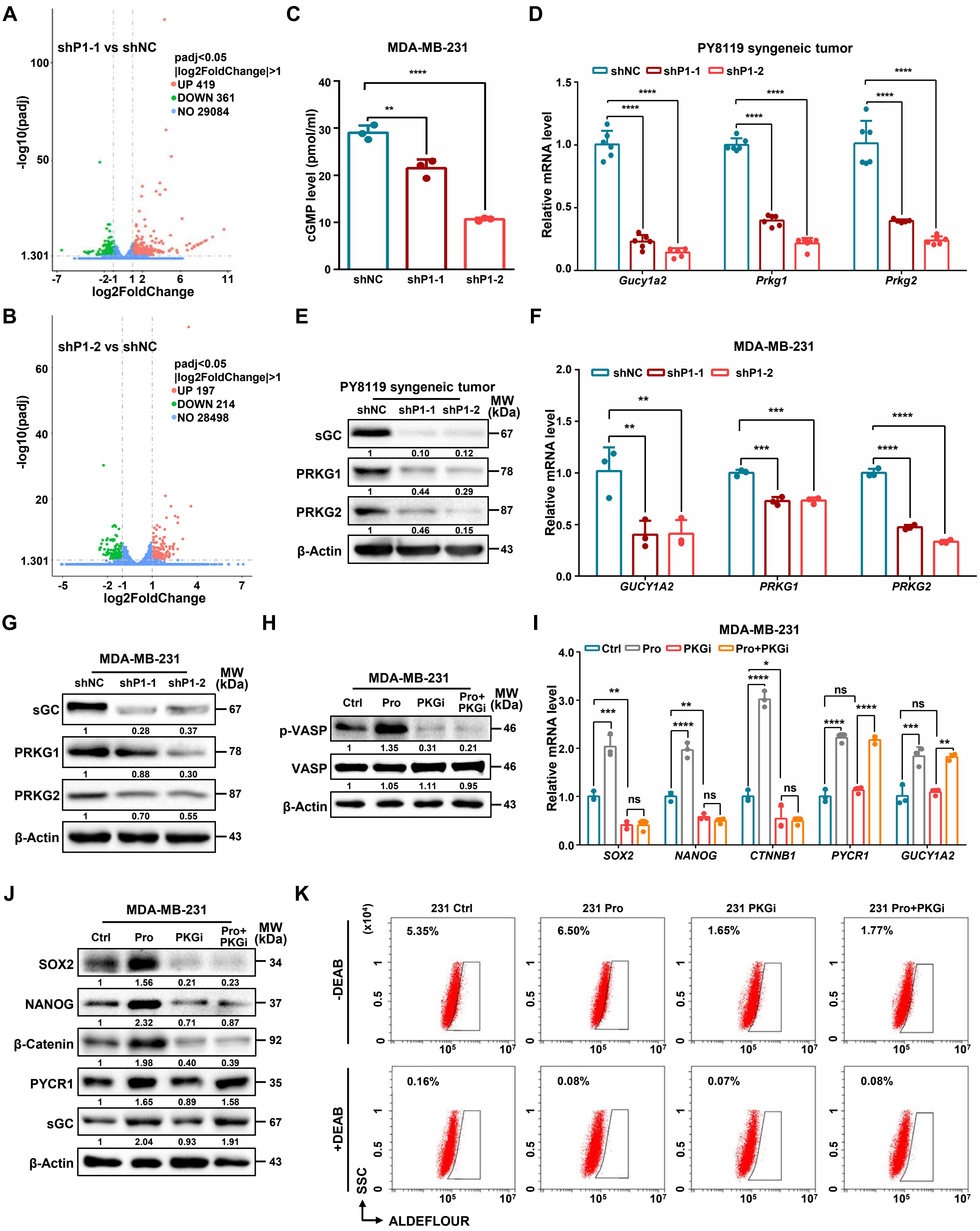

Supplementary Fig. 5

(**A-B**) Volcano plots displaying downregulated genes in MDA-MB-231 shP1-1 cells (**A**) and shP1-2 cells (**B**). The number of significantly variant genes (FC > 2, padj < 0.05) was shown. Vertical dashed lines indicate cut-off of FC (2), whereas the horizontal dashed lines indicate cut-off of P value (0.05). (**C**) The cGMP levels were measured following PYCR1 knockdown in MDA-MB-231 cells (n = 3). (**D**) Relative mRNA levels of *Gucy1a2*, *Prkg1* and *Prkg2* were determined following PYCR1 knockdown in PY8119 tumor (n = 6). (**E**) The protein levels of sGC, PRKG1 and PRKG2 were determined following PYCR1 knockdown in PY8119 tumor. (**F**) Relative mRNA levels of *GUCY1A2*, *PRKG1* and *PRKG2* were determined following PYCR1 knockdown in MDA-MB-231 cells (n = 3). (**G**) The protein levels of sGC, PRKG1 and PRKG2 were determined following PYCR1 knockdown in MDA-MB-231 cells. (**H**) The phosphorylation level of VASP (Ser239) was determined following treatment proline and PKGi in MDA-MB-231 cells. (**I**) Relative mRNA levels of *SOX2*, *NANOG*, *CTNNB1*, *PYCR1* and *GUCY1A2* were determined following treatment proline and PKGi in MDA-MB-231 cells (n = 3). (**J**) Relative protein levels of SOX2, NANOG, β-Catenin, PYCR1 and sGC were determined following treatment proline and PKGi in MDA-MB-231 cells. (**K**) Flow cytometry analysis for ALDH-positive cells in MDA-MB-231 cells treated proline and PKG inhibitor. PKGi, PKG inhibitor. Graph data were presented as mean ± SD. **P* < 0.05, ***P* < 0.01, ****P* < 0.001, *****P* < 0.0001. *P* values were calculated with two-tailed, unpaired Student's t-test (**C**, **D**, **F**) and one-way ANOVA (**I**).

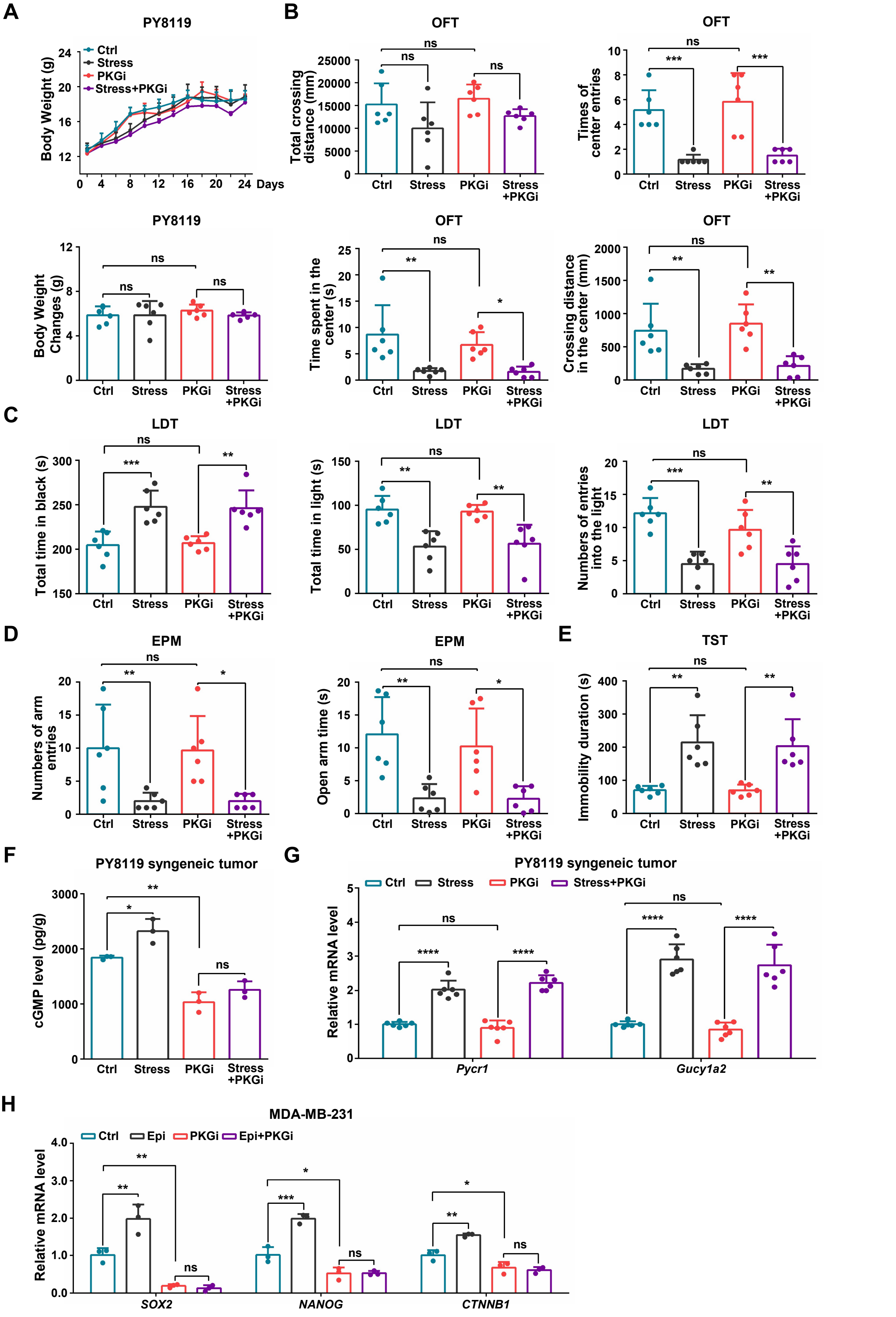

Supplementary Fig. 6

(**A**) Body weight changes of Ctrl, Stress, PKGi and Stress+PKGi PY8119 tumor in mice (n = 6). (**B**) Total crossing distance, times of center entries, time spent in the center and crossing distance in the center were analyzed in OFT (n = 6). (**C**) Total times in black, total time in light and numbers of entries into the light were analyzed in LDT (n = 6). (**D**) Numbers of arm entries and open arm times were analyzed in EPM (n = 6). (**E**) Immobility duration was analyzed in TST (n = 6). (**F**) The cGMP levels of PY8119 tumor (n = 3). (**G**) Relative mRNA levels of *Pycr1* and *Gucy1a2* were determined in PY8119 tumor (n = 3). (**H**) Relative mRNA levels of *SOX2*, *NANOG* and *CTNNB1* were determined following Epi and PKGi treatment in MDA-MB-231 cells (n = 3). OFT, open-field tests. LDT, light–dark box test. EPM, elevated plus maze test. TST, tail suspension test. Graph data were presented as mean ± SD. *P < 0.05, **P < 0.01, ***P < 0.001, ****P < 0.0001. P values were calculated with one-way ANOVA.

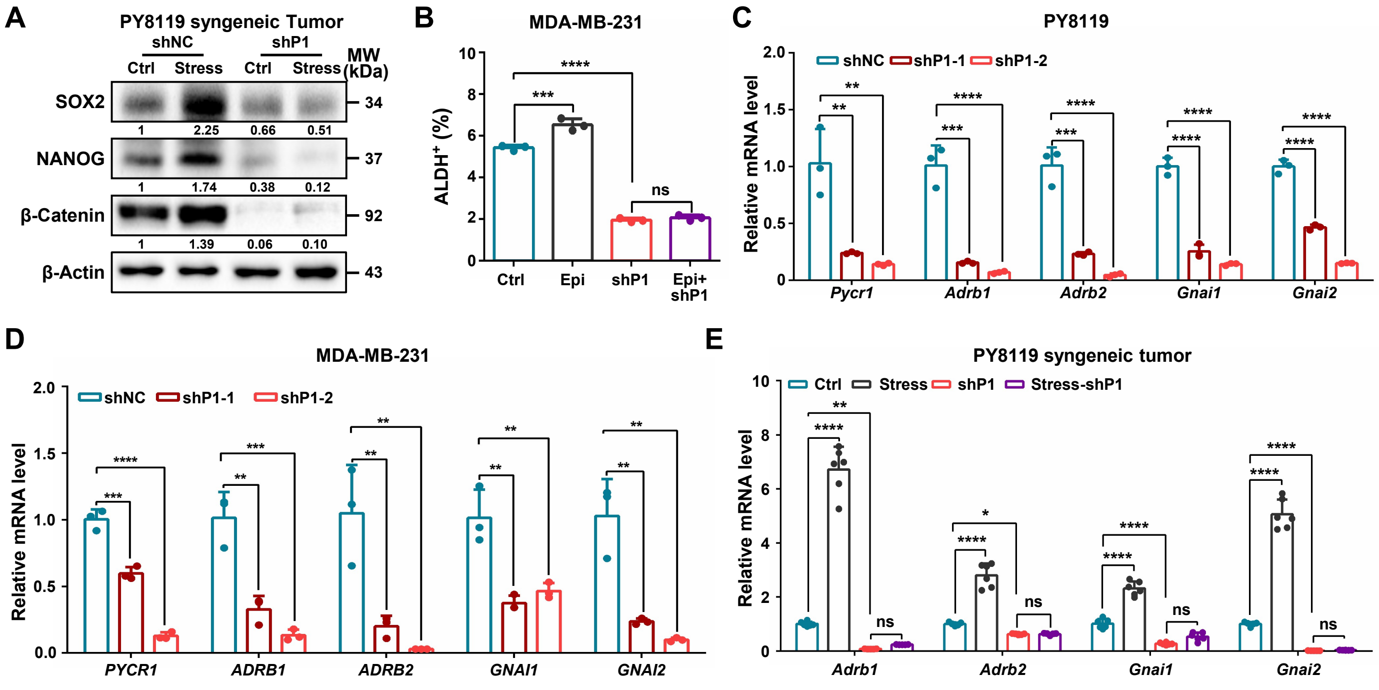

Supplementary Fig. 7

(**A**) Relative protein levels of stemness factors in PY8119 tumor. (**B**) Differences of ALDH-positive cells following PYCR1 knockdown and Epi treatment in MDA-MB-231 cells were analyzed (n = 3). (**C**-**D**) Relative mRNA levels of *PYCR1*, β-receptors (*ADRB1* and *ADRB2*) and G protein (*GNAI1* and *GNAI2*) related genes were determined following PYCR1 knockdown in PY8119 cells (**C**) and MDA-MB-231 cells (**D**) (n = 3). (**E**) Relative mRNA levels of β-receptors (*Adrb1* and *Adrb2*) and G protein (*Gnai1* and *Gnai2*) related genes were determined in PY8119 tumor (n = 6). Graph data were presented as mean ± SD. **P* < 0.05, ***P* < 0.01, ****P* < 0.001, *****P* < 0.0001. *P* values were calculated with two-tailed, unpaired Student's t-test (**C**, **D**) or one-way ANOVA (**B**, **E**).

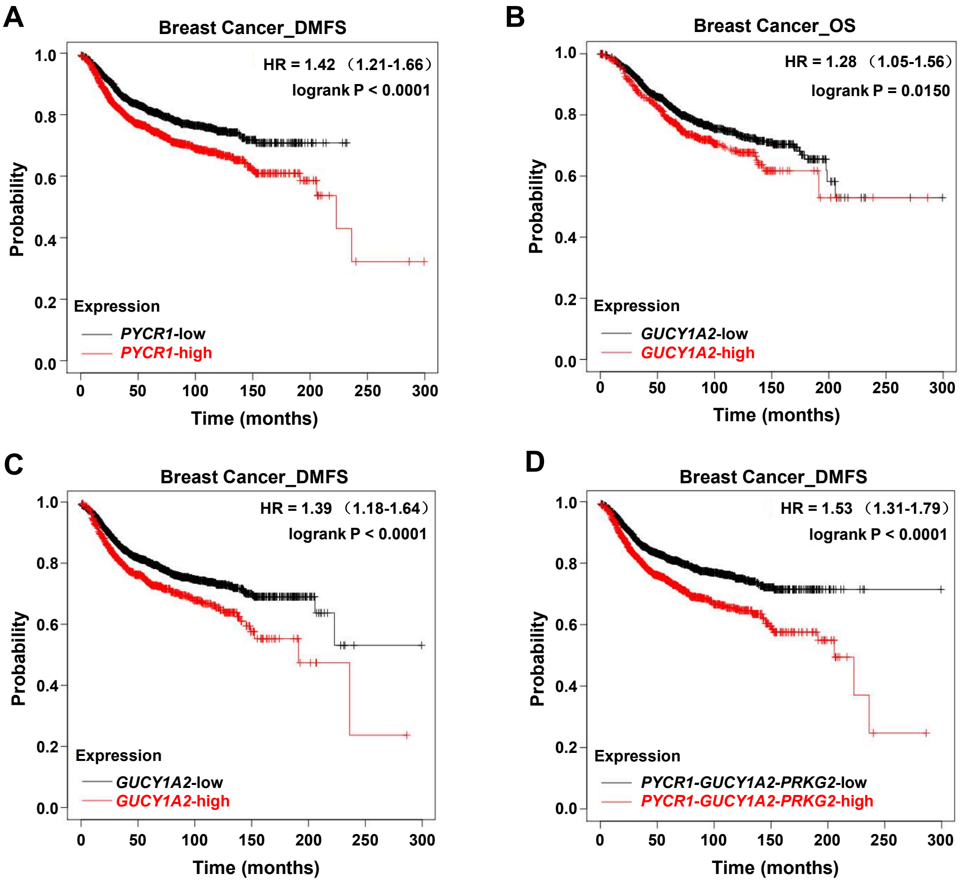

Supplementary Fig. 8

(**A**) Kaplan-Meier distant metastasis-free survival (DMFS) plots of breast cancer patients created using Kaplan-Meier Plotter network. Patients were classified into *PYCR1* high and *PYCR1* low and analyzed as indicated. (**B-C**) Kaplan-Meier overall survival (OS) (**B**) and distant metastasis-free survival (**C**) plots of breast cancer patients created using Kaplan-Meier Plotter network. Patients were classified into *GUCY1A2* high and *GUCY1A2* low subgroups, and analyzed as indicated. (**D**) Kaplan-Meier distant metastasis-free survival plots of breast cancer patients created using Kaplan-Meier Plotter network. Patients were classified into *PYCR1-GUCY1A2-PRKG2* high and *PYCR1-GUCY1A2-PRKG2* low subgroups and analyzed as indicated.

Primers of shRNAs	Sense (5'-3')	Antisense (5'-3')
**shNC**	TTCTCCGAACGTGTCACGT	ACGTGACACGTTCGGAGAA
**Human-shPYCR1-1**	CACAGTTTCTGCTCTCAGGAA	CACAGTTTCTGCTCTCAGGAA
**Human-shPYCR1-2**	GCCCACAAGATAATGGCTA	GCCCACAAGATAATGGCTA
**Mus-shPycr1-1**	GGCTTTCGCTCCTTGCTTATC	GGCTTTCGCTCCTTGCTTATC
**Mus-shPycr1-2**	GCCAAGATGCTACTAGACTCA	GCCAAGATGCTACTAGACTCA
**Primers of plasmid construction**	**Sense (5'-3')**	**Antisense (5'-3')**
**pLVX-PYCR1**	CGGAATTCGCCACCATGAGCGTGGGCT TCATCGGCGC	CGGGATCCCGATCCTTGCCCGCTGGGGCCA

Supplementary Table 1: Sequences of primers for plasmid construction

Primers of RT-qPCR	Sense (5′-3′)	Antisense (5′-3′)
**Human-PYCR1**	TGGCTGCCCACAAGATAATGG	TCCTCAATGTCGGCGCCTATT
**Human-PYCR2**	AAGCCAGACACATCGTGGTC	CGTACACTGTAGCGCCTTC
**Human-PYCR3**	CTCATCAGAGCAGGAAAAGTG	TTGGGCAAGACCCGC
**Human-SOX2**	TGGACAGTTACGCGCACAT	CGAGTAGGACATGCTGTAGGT
**Human-NANOG**	ACCTATGCCTGTGATTTGTGG	AGTGGGTTGTTTGCCTTTGG
**Human-CTNNB1**	ATGGAGCCGGACAGAAAAGC	TGGGAGGTGTCAACATCTTCTT
**Human-ACTB**	TTGCCGACAGGATGCAGAAGGA	AGGTGGACAGCGAGGCCAGGAT
**Human-PRKG1**	GGACAGGACTCATCAAGCATAC	CTTCACGAGTGACATTTACCGTT
**Human-PRKG2**	ATCCCAACTCCACCATTTCCT	GTACAATTCTGCATACAGATACCAA
**Human-GUCY1A2**	TGCTGGGGATGATTAAGGCTG	GTTGATGCTAATTCTGAGGTCCG
**Human-ADRB1**	ATCGAGACCCTGTGTGTCATT	GTAGAAGGAGACTACGGACGAG
**Human-ADRB2**	TTGCTGGCACCCAATAGAAGC	CAGACGCTCGAACTTGGCA
**Human-GNAI1**	TTAGGGCTATGGGGAGGTTGA	GGTACTCTCGGGATCTGTTGAAA
**Human-GNAI2**	TACCGGGCGGTTGTCTACA	GGGTCGGCAAAGTCGATCTG
**Mus-Pycr1**	ATGAGCGTAGGCTTCATCGG	GTGTCAGGTTCACCCCTATCT
**Mus-Sox2**	CGGCACAGATGCAACCGAT	CCGTTCATGTAGGTCTGCG
**Mus-Nanog**	CACAGTTTGCCTAGTTCTGAGG	GCAAGAATAGTTCTCGGGATGAA
**Mus-Ctnnb1**	ATGGAGCCGGACAGAAAAGC	TGGGAGGTGTCAACATCTTCTT
**Mus-Actb**	GAGGTATCCTGACCCTGAAGTA	CACACGCAGCTCATTGTAGA
**Mus-Prkg1**	ATCCGAGAGGTCGAAGGATCT	ATTCCACGGGGTACATACAGT
**Mus-Prkg2**	TGAAGAACGGGATCAACGAC	CGCTGTGTCACTGATTCTCA
**Mus-Gucy1a2**	GCTCTAGAGAGGCCGCTAA	GAATGGTCTGCATAGGAGCA
**Mus-Adrb1**	CTCATCGTGGTGGGTAACGTG	ACACACAGCACATCTACCGAA
**Mus-Adrb2**	ATGTCGGTTATCGTCCTGGC	GGTTTGTAGTCGCTCGAACTTG
**Mus-Gnai1**	GGTTTACAGACACGTCCATCAT	GCCTGCATATTCTGGGTAGCAT
**Mus-Gnai2**	CAGATCGACTTTGCTGATCCC	TAAGCGGCTGAGTCATTGAGC

Supplementary Table 2: Primers for RT-qPCR

Antibodies	Company	Catalog
**β–Catenin (Rabbit monoclonal)**	CST	Cat#8480<br>RRID:AB_11127855
**NANOG (Rabbit polyclonal)**	Abcam	Cat#ab80892<br>RRID:AB_2150114
**SOX-2 (E-4) (Mouse monoclonal)**	Santa Cruz Biotechnology	Cat#sc-365823<br>RRID:AB_10842165
**PYCR1(Rabbit polyclonal)**	Proteintech	Cat#13108-1-AP<br>RRID:AB_2174878
**PRKG1 (Rabbit polyclonal)**	Proteintech	Cat#21646-1-AP<br>RRID:AB_2878897
**GUCY1B3 (Rabbit polyclonal)**	Proteintech	Cat#19011-1-AP<br>RRID:AB_2115817
**VASP (Rabbit polyclonal)**	Proteintech	Cat#13472-1-AP<br>RRID:AB_2213418
**Anti-cGKII antibody (Rabbit polyclonal)**	Huabio	Cat#ER63000<br>RRID:AB_2925218
**Phospho-VASP (Ser239) (Rabbit polyclonal)**	Affinity	Cat#AF3338<br>RRID:AB_2834753
**β-Actin (Rabbit monoclonal)**	abclonal	Cat#AC038<br>RRID:AB_2863784
**Chemicals, peptides, and recombinant proteins**	Company	Catalog
**Mammary Epithelial Cell Growth Medium BulletKit**	Lonza/Clonetics Corporation	Cat#CC-3150
**Minimum Essential Medium**	Gibco	Cat#A1048901
**fetal bovine serum (FBS)**	Gibco	Cat#10270
**Leibovitz's L-15 Medium**	Gibco	Cat#11415064
**McCoy's 5a Medium**	Gibco	Cat#16600082
**RPMI-1640**	Gibco	Cat#C11875500BT
**F12K**	HyClone	Cat#SH30526.01
**Anti-mycoplasma reagent SaveIt**	Hanbio	Cat#HB-SV-1000
**Penicillinstreptomycin**	Thermo	Cat#15140122
**DMEM**	Gibco	Cat# C11995500BT
**Insulin**	Sigma,	Cat#91077C
**Matrigel**	Corning	Cat#354234
**Lipo2000**	Invitrogen	Cat#11668500
**Puromycin**	Sigma	Cat#P8833
**L-proline**	MCE	Cat#HY-Y0252
**KT5823**	MCE	Cat#HY-N6791
**Epinephrine bitartrate**	Selleck	Cat#S2521
**L-Glutamine**	Solarbio	Cat#G0200
**TRIzol**	Life Technologies	Cat#15596-026
**EasyScript One-Step gDNA Removal cDNA Synthesis SuperMix**	Transgen	Cat#AE311-03
**Evo M-MLV RT Kit with gDNA Clean for qPCR**	ACCURATE BIOTECHNOLOGY	Cat#AG11705
**2X Universal SYBR Green Fast qPCR Mix**	ABclonal	Cat#RK21203
**ChamQTM Universal SYBR qPCR Master Mix**	Vazyme	Cat#Q711-02
**protease inhibitor cocktail**	MCE	Cat#HY-K0010
**Phosphatase Inhibitor Cocktail**	Bimake	Cat#B15001
**PageRuler Prestained Protein Ladder**	Thermo Fisher Scientific	Cat#26617
**ECL kits**	Thermo Fisher Scientific	Cat#34580
**SPlink Detection Kits**	ZSGB-BIO	Cat#SP9000
**ALDEFLUOR Kit**	STEMCELL Technologies	Cat#01700
**DMEM/F12 medium**	Gibco	Cat#C11330500BT
**methylcellulose**	R&D Systems	Cat#HSC001
**basic fibroblast growth factor**	Peprotech	Cat#100-18B
**B-27**	Gibco	Cat#17504044
**epidermal growth factor**	Sigma	Cat#E9644
**proline assay kit**	Solarbio	Cat#BC0295
**cGMP ELISA kit**	Cloud-Clone Corp	Cat#CEA577Ge
**Bovine serum albumin (BSA)**	Sigma	Cat#V900933
**Annexin V-EGFP/PI kit**	Abbkine	Cat#KTA0005
**Cell Counting Kit-8**	Meilunbio	Cat#MA0218
**Dnase Ⅰ**	Merck	Cat#10104159001
**Collagenase Ⅰ**	Gibco	Cat#17018029

Supplementary Table 3: Reagents
