# Supplementary material for: Pyrroline-5-carboxylate reductase 1 reprograms proline metabolism to drive breast cancer stemness under psychological stress
Source: Cell Death Dis. 2023 Oct 16;14(10):682. doi: 10.1038/s41419-023-06200-5 (PMC10579265; doi:10.1038/s41419-023-06200-5)

Full and uncropped western blots for Fig. 2D

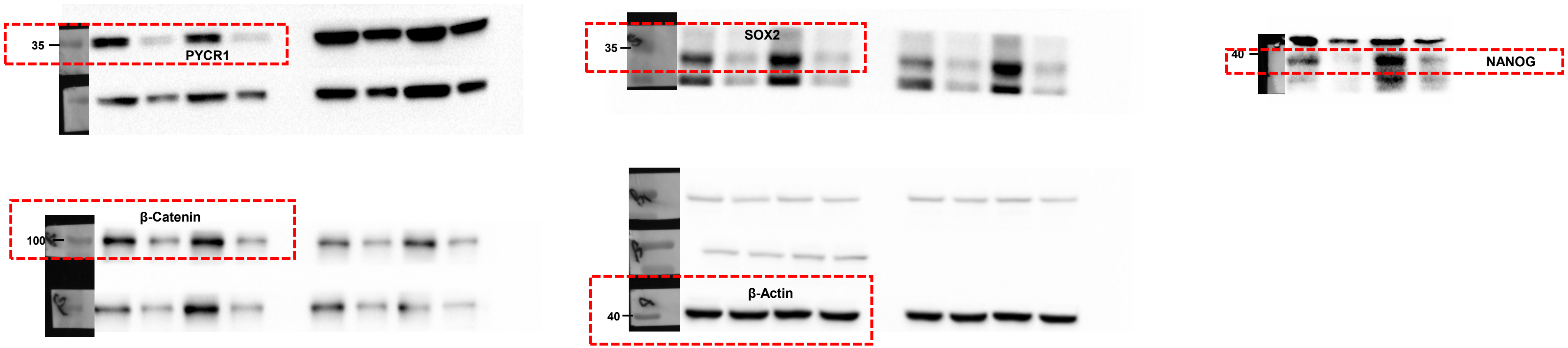

Full and uncropped western blots for Fig. 2G

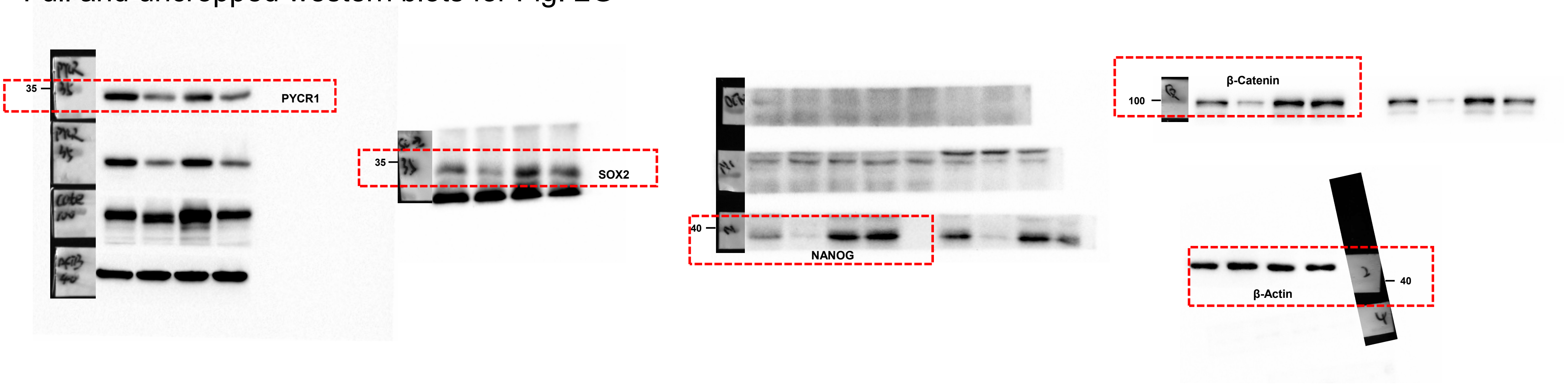

Full and uncropped western blots for Fig. 2L

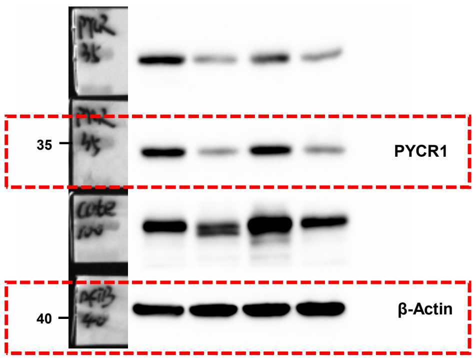

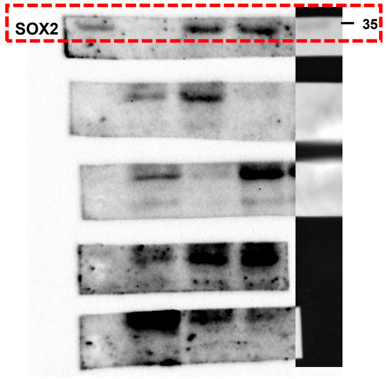

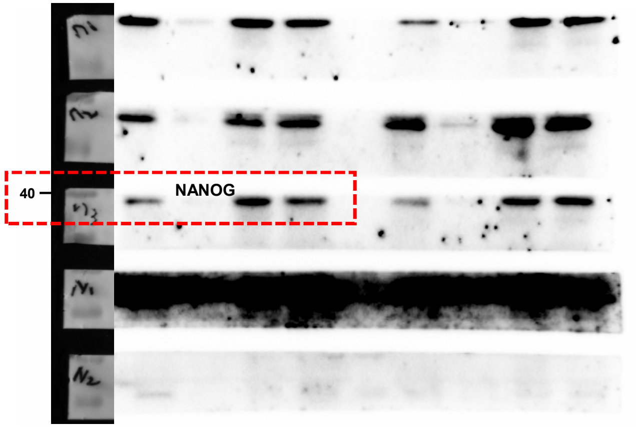

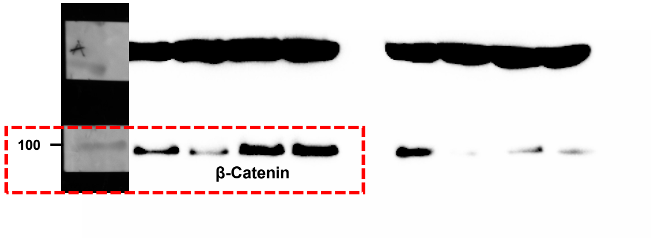

Full and uncropped western blots for Fig. 3F

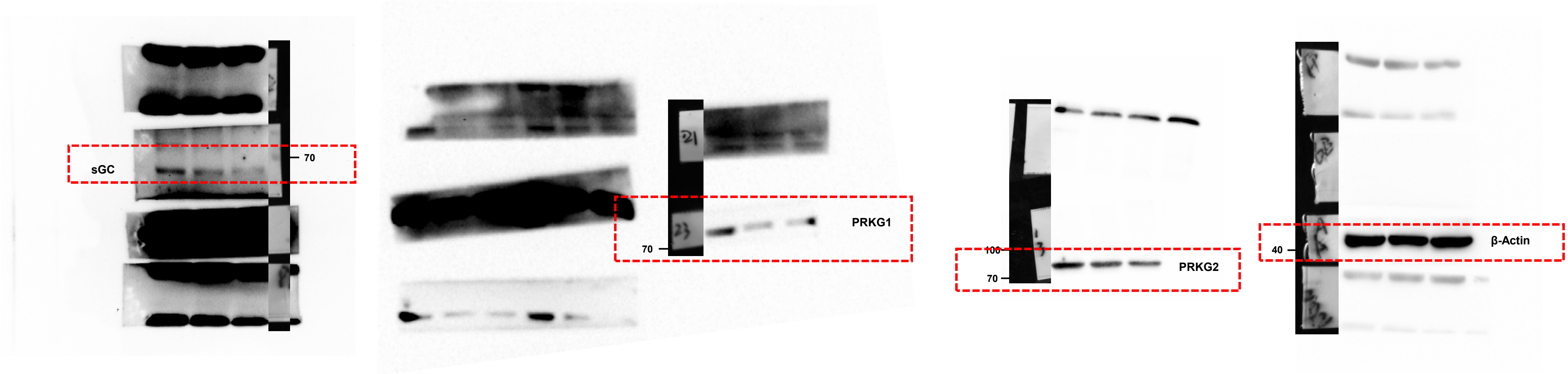

Full and uncropped western blots for Fig. 3I

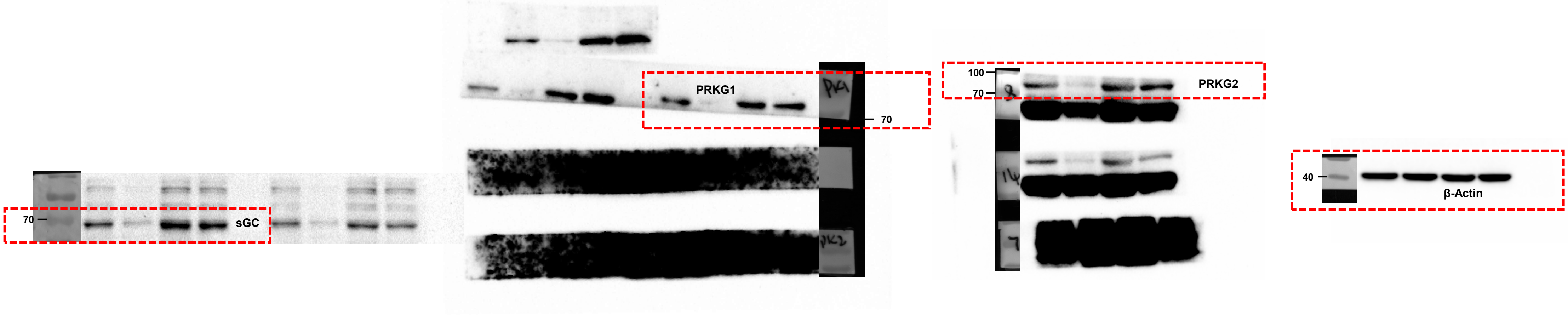

Full and uncropped western blots for Fig. 4D

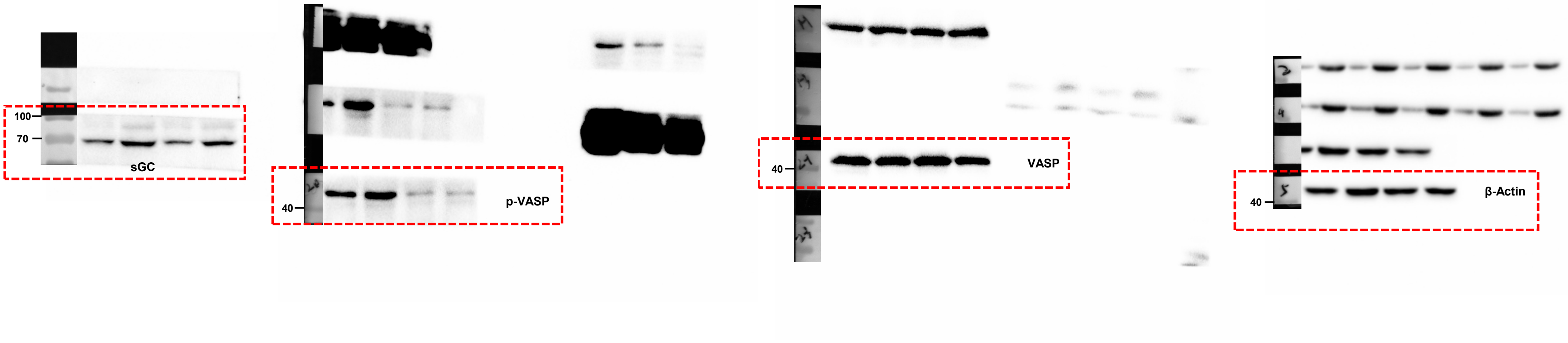

Full and uncropped western blots for Fig. 4G

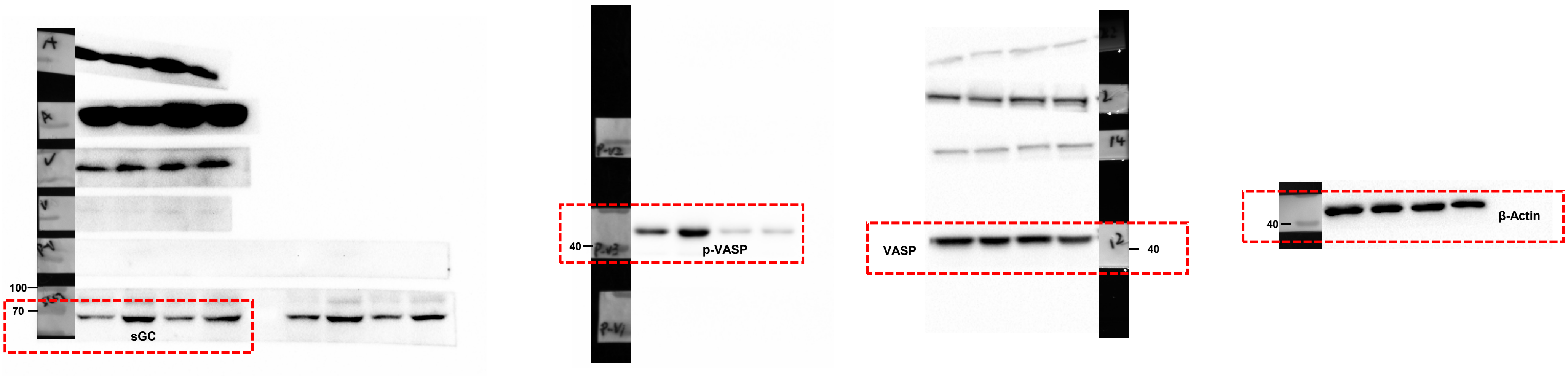

Full and uncropped western blots for Fig. 5G

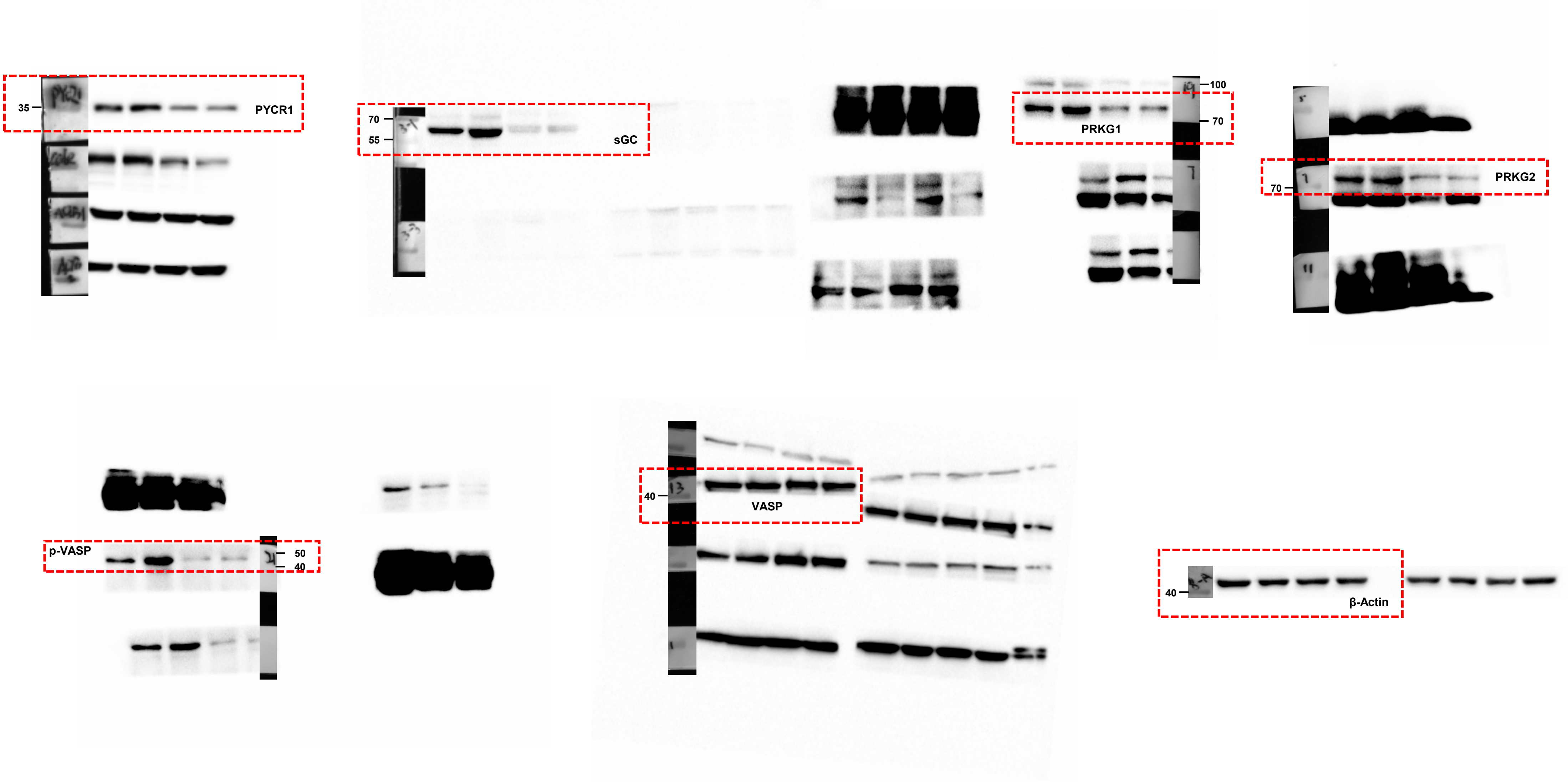

Full and uncropped western blots for Fig. 6D

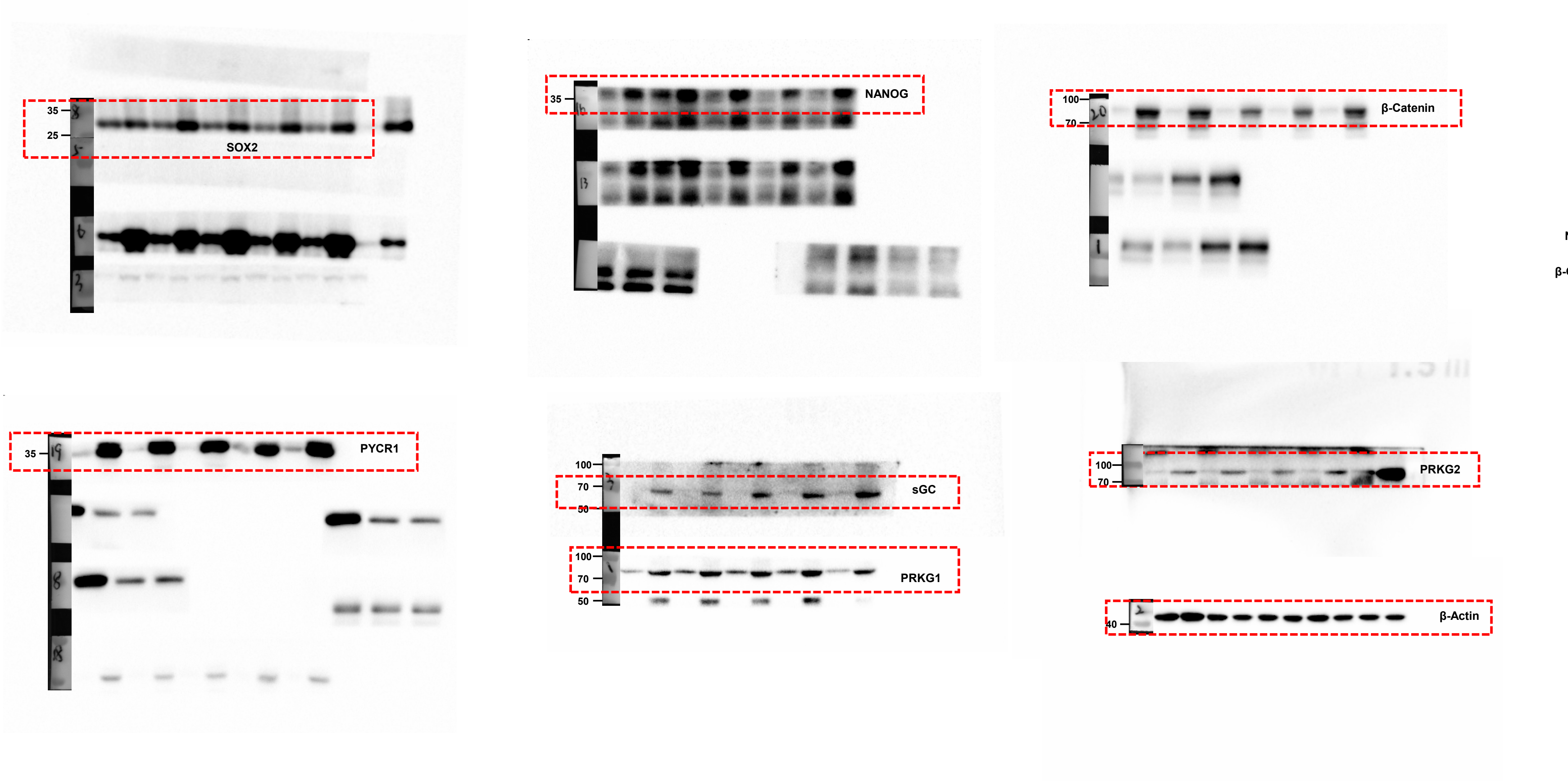

Full and uncropped western blots for Supplementary Fig. 1J

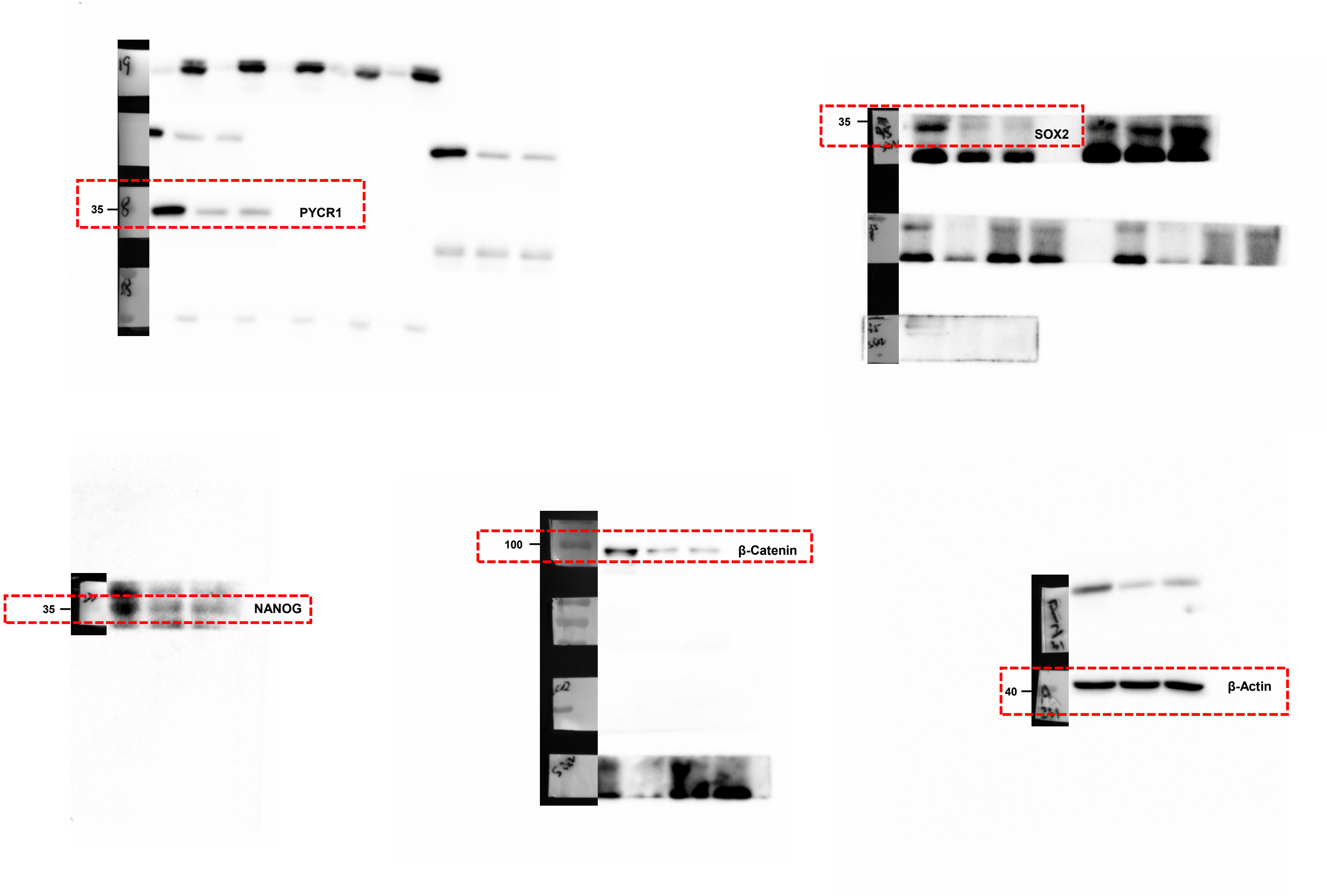

Full and uncropped western blots for Supplementary Fig. 2C

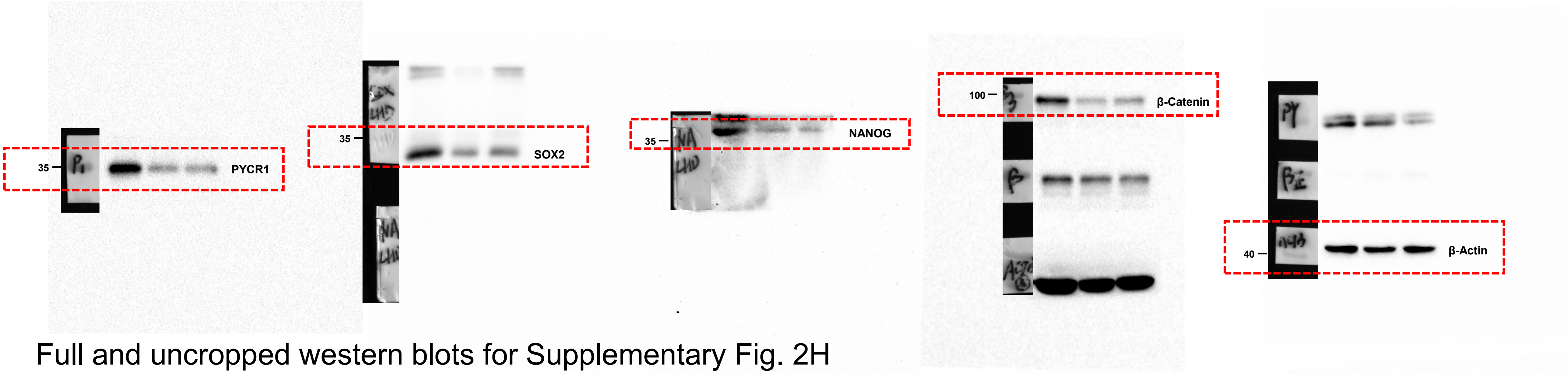

Full and uncropped western blots for Supplementary Fig. 2H

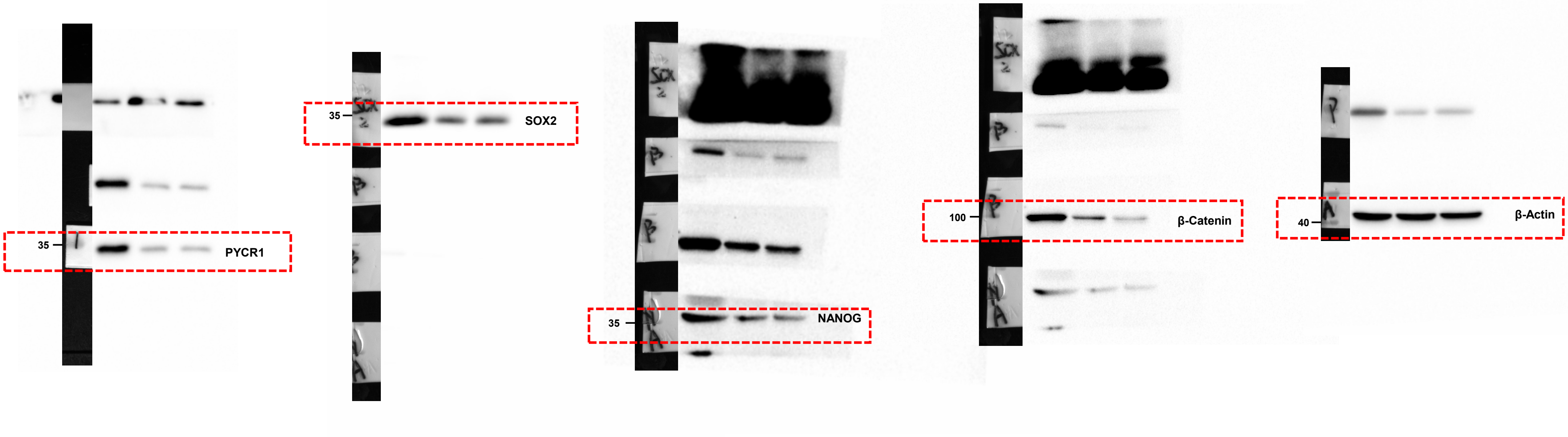

Full and uncropped western blots for Supplementary Fig. 3B

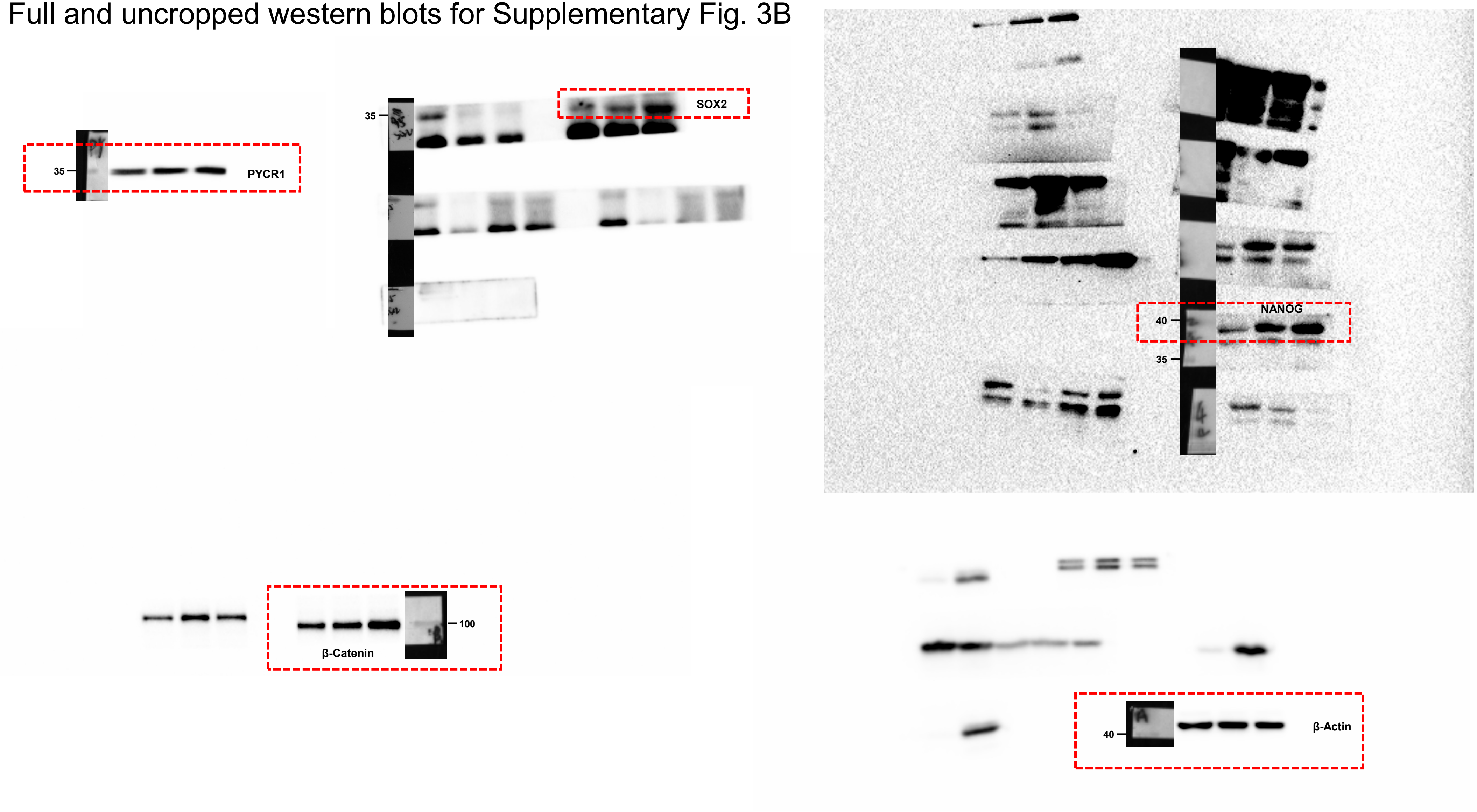

Full and uncropped western blots for Supplementary Fig. 5E

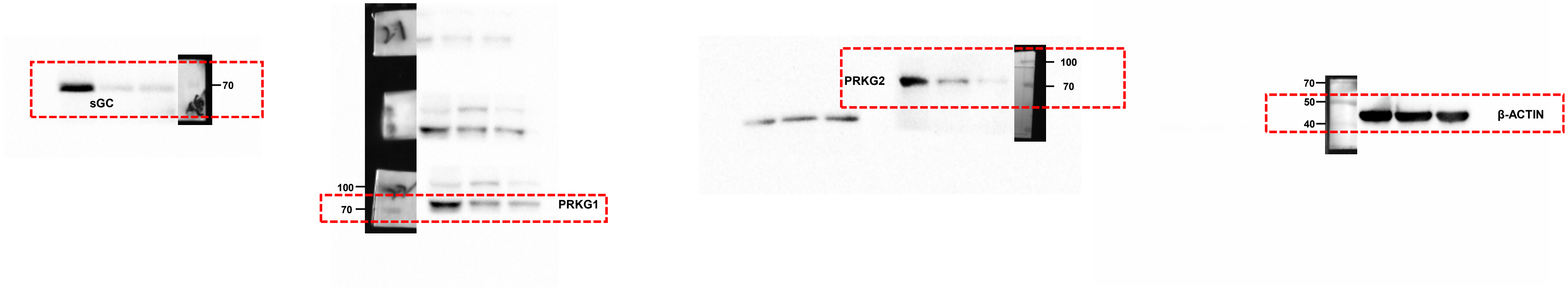

Full and uncropped western blots for Supplementary Fig. 5G

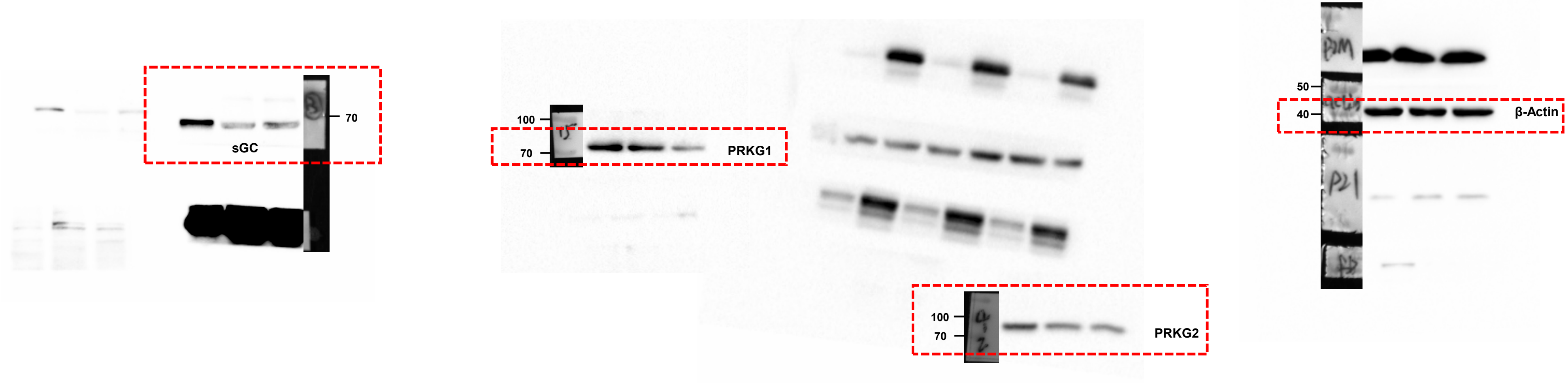

Full and uncropped western blots for Supplementary Fig. 5H

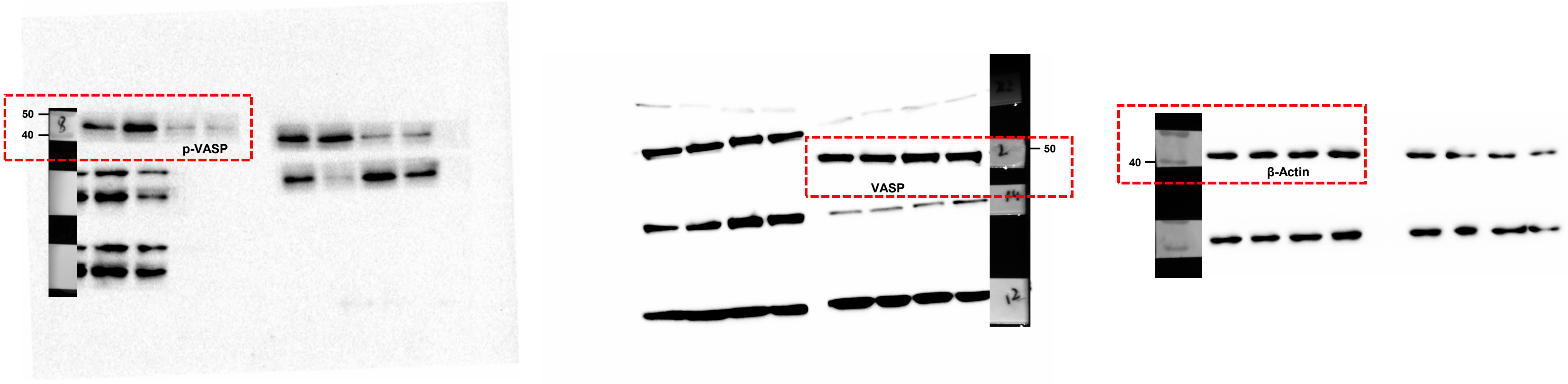

Full and uncropped western blots for Supplementary Fig. 5J

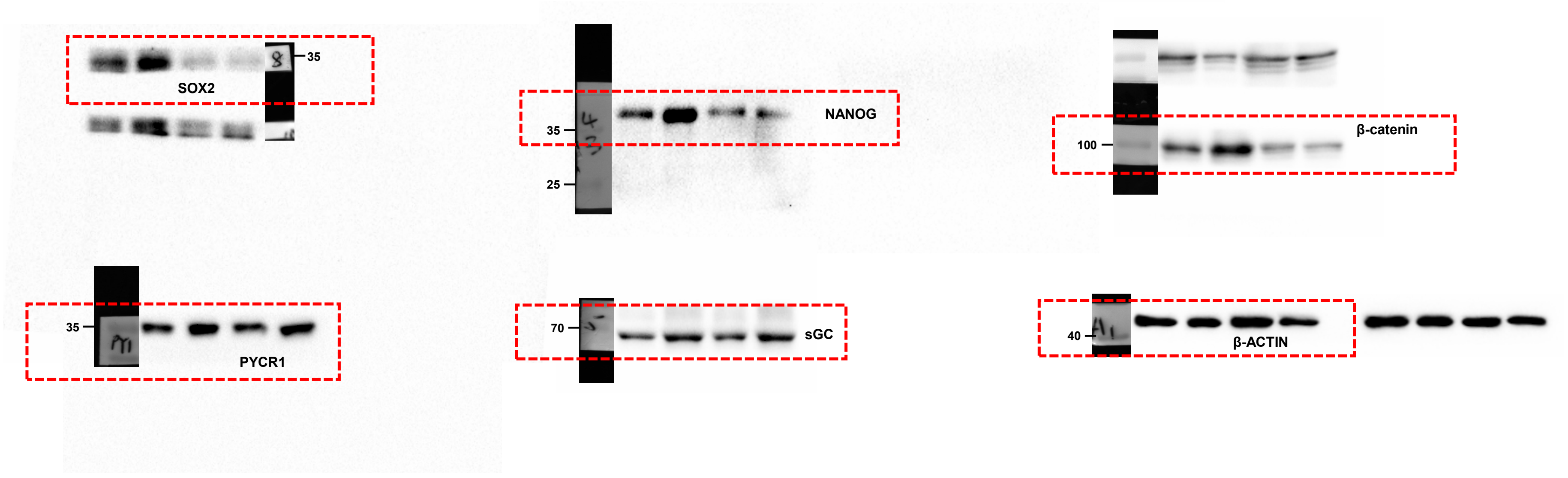

Full and uncropped western blots for Supplementary Fig. 7A

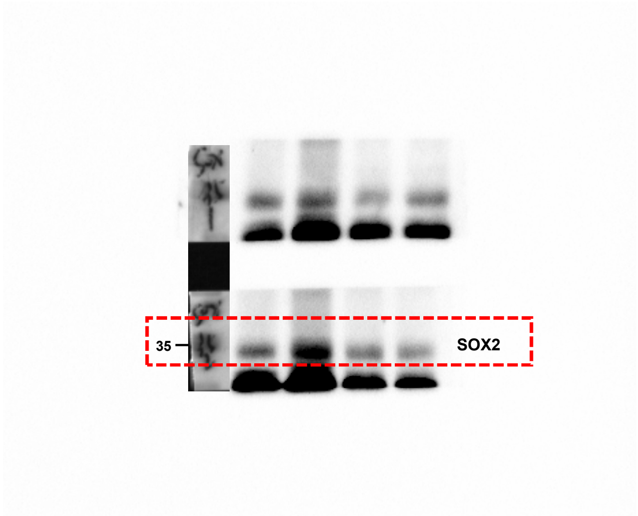

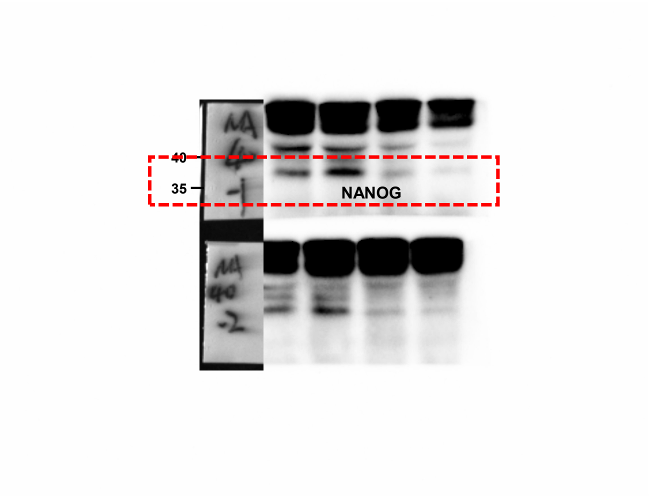

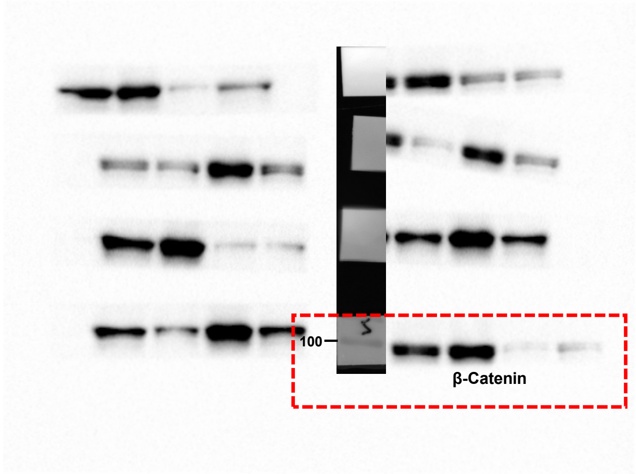

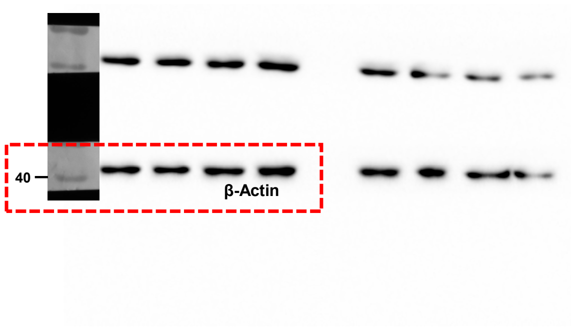

Supplement: Supplementary file 2 — Original Data File [file 41419_2023_6200_MOESM2_ESM.pdf]
